# Striking variation in chromosome structure within *Musa acuminata* subspecies, diploid cultivars, and F1 diploid hybrids

**DOI:** 10.3389/fpls.2024.1387055

**Published:** 2024-07-04

**Authors:** Denisa Beránková, Jana Čížková, Gabriela Majzlíková, Alžběta Doležalová, Hassan Mduma, Allan Brown, Rony Swennen, Eva Hřibová

**Affiliations:** ^1^ Institute of Experimental Botany of the Czech Academy of Sciences, Centre of Plant Structural and Functional Genomics, Olomouc, Czechia; ^2^ International Institute of Tropical Agriculture, Banana Breeding, Arusha, Tanzania; ^3^ International Institute of Tropical Agriculture, Kampala, Uganda; ^4^ Division of Crop Biotechnics, Laboratory of Tropical Crop Improvement, Katholieke Universiteit Leuven, Leuven, Belgium

**Keywords:** oligo painting FISH, chromosome translocation, comparative cytogenetics, *Musa acuminata*, F1 hybrids

## Abstract

The majority of cultivated bananas originated from inter- and intra(sub)specific crosses between two wild diploid species, *Musa acuminata* and *Musa balbisiana*. Hybridization and polyploidization events during the evolution of bananas led to the formation of clonally propagated cultivars characterized by a high level of genome heterozygosity and reduced fertility. The combination of low fertility in edible clones and differences in the chromosome structure among *M. acuminata* subspecies greatly hampers the breeding of improved banana cultivars. Using comparative oligo-painting, we investigated large chromosomal rearrangements in a set of wild *M. acuminata* subspecies and cultivars that originated from natural and human-made crosses. Additionally, we analyzed the chromosome structure of F1 progeny that resulted from crosses between Mchare bananas and the wild *M. acuminata* ‘Calcutta 4’ genotype. Analysis of chromosome structure within *M. acuminata* revealed the presence of a large number of chromosomal rearrangements showing a correlation with banana speciation. Chromosome painting of F1 hybrids was complemented by Illumina resequencing to identify the contribution of parental subgenomes to the diploid hybrid clones. The balanced presence of both parental genomes was revealed in all F1 hybrids, with the exception of one clone, which contained only Mchare-specific SNPs and thus most probably originated from an unreduced diploid gamete of Mchare.

## Introduction

Edible banana clones are an important trade commodity in tropical and subtropical countries and a staple food crop in Eastern Africa. They have originated by natural intrasubspecific and interspecific hybridization, and polyploidization in some cases. Most edible bananas originated from crosses between two wild *Musa* species, *M. acuminata* (donor of A genome) and *M. balbisiana* (donor of B genome) (Simmonds and Shepherd, 1955), with the possible contribution of other *Musa* species (Martin et al., 2020a; Sardos et al., 2022). Both species have small genomes and contain the same number of chromosomes (2*n* = 22). Diversity studies showed higher variability in genome sizes as well as higher genetic variability within *M. acuminata*, which contains several subspecies, compared to *M. balbisiana* (Janssens et al., 2016; Sardos et al., 2016a, 2020; Christelová et al., 2017). Domestication of bananas began in the Holocene, around 7,000 years bp, in Southeast Asia, when human migration brought banana species and subspecies from different regions into close proximity and therefore enabled natural crosses (Perrier et al., 2011).

Hybridization and polyploidization events during banana evolution led to the formation of parthenocarpic, clonally propagated cultivars, which are characterized by a high level of heterozygosity. Seven different translocation groups among *M. acuminata* were described by Shepherd (1999), who studied chromosome pairing during meiosis. As was recently shown, some of these large chromosomal translocations are characteristic of individual wild diploid species or even subspecies of *M. acuminata* (Baurens et al., 2019; Martin et al., 2017; Šimoníková et al., 2019; Dupouy et al., 2019; Martin et al., 2020b, 2020; Liu et al., 2023). Chromosome structural heterozygosity is common and results in aberrant chromosome pairing during meiosis and reduced or even zero production of fertile gametes (Dodds and Simmonds, 1948; Fauré et al., 1993; Shepherd, 1999; Goigoux et al., 2013). The reduced fertility of edible banana cultivars, together with differences in chromosome structure among *M. acuminata* subspecies greatly hampers the breeding of improved banana cultivars (Batte et al., 2019; Baurens et al., 2019) with lower susceptibility to diseases and pests. Diseases in commercial plantations are currently being controlled by the frequent application of fungicides; however, this treatment has a negative effect on the environment and health of banana workers (De Bellaire et al., 2010; Cordoba and Jansen, 2014).

Nowadays, the most-grown groups of bananas are triploid cultivars, including dessert bananas such as Cavendish and Gros Michel (AAA genome), cooking East African Highland bananas (AAA genome), and plantains (AAB genome). The Cavendish subgroup of bananas itself is the world’s most exported fruit, with an annual global export quantity of about 20 million tons (FAO, 2023). Several recent studies focused on the characterization of parentage relationships of selected diploid and triploid edible banana clones, including the triploid sweet banana cultivar Cavendish, showing the contribution from Mchare bananas (Raboin et al., 2005; Perrier et al., 2009; Martin et al., 2023b). The Mchare cultivar subgroup forms a phenotypically distinct group of diploid (AA genome) cooking bananas, which are nowadays found only in East Africa and some East African Islands. The study of Martin et al. (2023b) showed that the Mchare bananas contributed the unreduced 2*x* gamete to the origin of Cavendish bananas and the closely related Gros Michel cultivar subgroup (Martin et al., 2023b). The identification of Mchare bananas as the direct parents of successful cultivars highlights the importance of their use in breeding programs and warrants a more detailed analysis of their genome structure in the landraces and their hybrid offspring.

Improved triploid banana cultivars can be obtained through the development of tetraploids (4*x*), followed by the production of secondary triploid hybrids (Bakry and Horry, 1992; Tomekpe et al., 2004; Ortiz, 2013; Ortiz and Swennen, 2014; Nyine et al., 2017). Improved diploid varieties are produced by crosses of the existing cultivars with improved diploids (2*x* × 2*x* crosses). Traditional breeding requires the production of 4*x* or improved 2*x* genotypes that produce seeds, followed by re-establishing seed-sterile end products. Unfortunately, this process is hampered by the almost complete sterility of edible cultivars and flower incompatibility when stigma development does not correlate with bract lifting (Amah et al., 2021) and fast loss of pollen viability (Bayo et al., 2024). In combination with limited information on genome structural heterozygosity, the breeding process leads to very low seed sets. For instance, four seeds per Matooke (genome AAA) and only 1.6 seeds per Mchare (genome AA) can be obtained on average from one bunch (Brown et al., 2017; Batte et al., 2019).

Large genome structural changes can be identified by new sequencing technologies that span large sequence regions, e.g., mate-pairs Illumina sequences or long-read sequences produced by PacBio or Oxford Nanopore Technologies (Baurens et al., 2019; Dumschott et al., 2020; Pucker et al., 2022). Molecular cytogenetic techniques represent another option for the identification of large genome rearrangements. Localization of whole chromosome painting probes onto mitotic chromosomes *in situ* provides a powerful tool to identify large chromosomal translocations and to perform comparative analysis of chromosome structure in plants (e.g., Braz et al., 2018; Hou et al., 2018; Yu et al., 2022). Oligo-painting fluorescence *in situ* hybridization (FISH) is based on the *in silico* identification of large sets of unique oligomers specific to genome/chromosomal regions of interest (reviewed in Jiang, 2019) that can serve as probes. In bananas, chromosome-arm-specific sets of 45-nt-long sequences have already been identified and used as probes for *in situ* localization (Šimoníková et al., 2019). It has been shown that banana-specific painting probes designed based on the reference genome sequence of *M. acuminata* ssp. *malaccensis* ‘DH Pahang’, can be used to study genome rearrangements in other banana species and subspecies, including those that play an important role in the evolution of most edible cultivars (Šimoníková et al., 2020). Their use permitted the identification of general chromosome structures in different *M. acuminata* (A genome) subspecies, *M. balbisiana* (B genome) and *M. schizocarpa* (S genome), and most importantly, in edible banana clones (Šimoníková et al., 2020). These results showed the presence of specific chromosome structures in different species and subspecies. Furthermore, the presence of translocation events detected only in one chromosome set in some wild diploid species (e.g., *M. acuminata* ssp. *siamea* ‘Pa Rayong’, and *M. acuminata* ssp. *burmannica* ‘Tavoy’) was observed and indicated their hybrid origin (Šimoníková et al., 2020).

Our present study is focused on the characterization of large chromosomal translocations in a set of wild *M. acuminata* subspecies and edible banana cultivars that originated from natural intrasubspecific crosses. One of the main aims was to provide insight into the chromosomal evolution of diploid banana species and their cultivars and to identify variability and mode of the genome rearrangements. In addition to natural cultivars, we analyzed the chromosome structure of F1 progeny that resulted from crosses between Mchare bananas (the ancestor of Cavendish and Gros Michel) and the wild *M. acuminata* ssp*. burmannicoides* ‘Calcutta 4’ genotype. As Mchare bananas were found to be of hybrid origin, we wanted to analyze if some of the chromosome structures were preferentially transmitted to the progeny. Comparative chromosome painting revealed large variations in the genome structure within *M. acuminata* cultivars and the presence of translocation events, which were not observed in wild species analyzed so far. Our findings support previous assumptions about a more complex mode of *Musa acuminata* evolution and also show that the origin of edible banana clones was most probably accompanied by repeated introgressions and backcrosses (De Langhe et al., 2010; Martin et al., 2023a).

## Materials and methods

### Plant material and diversity tree construction

Most *Musa* genotypes were obtained from the International *Musa* Transit Centre (ITC, Bioversity International, Leuven, Belgium) as *in vitro* plantlets, transferred to soil, and kept in a greenhouse. Root tips of Mchare clones and their F1 hybrids were collected and fixed from the plants stored at a field collection of the International Institute of Tropical Agriculture (IITA), NM-AIST, Arusha, Tanzania. The accessions used in the current study are listed in **Table 1**.

**Table 1:** List of *Musa* accessions analyzed in this work.

Species	Subspecies/subgroup	Accession name	ITC code^a^	Genbank DOI	Genomic constitution	Chromosome number (2*n*)
*M. acuminata*	*banksii*	‘Banksii’	0341	10.18730/9JSM$	AA	22
	*banksii*	‘Higa’	0428	10.18730/9JXPG	AA	22
	*banksii*	–	0896	10.18730/9KSY*	AA	22
	*malaccensis*	–	1886	10.18730/P5GEA	AA	22
	*malaccensis*	–	1887	10.18730/SAK1W	AA	22
	*zebrina*	‘Zebrina’	1139	10.18730/9M6P~	AA	22
	*siamea*	‘Khae (Phrae)’	0660	10.18730/9KCD6	AA	22
Cultivars	Unknown	‘Tuu Gia’	0610	10.18730/9K95D	AA	22
	–	‘Himone’	0886	10.18730/9KS68	AA	22
	–	‘Maleb’	0809	10.18730/9KKRK	AA	22
	–	‘Marakudu’	1210	10.18730/9MB6X	AA	22
	–	‘Vudu Beo’	1211	10.18730/9MB8Z	AA	22
	Sucrier	‘Mai’a hapai’	1172	10.18730/9M8EF	AA	22
	Rose	‘Rose’	0712	10.18730/9KER7	AA	22
	Mchare	‘Kahuti’	–	–	AA	22
	Mchare	‘Mchare mlelembo’	–	–	AA	22
	Mchare	‘Mchare laini’	–	–	AA	22
F1 hybrids						
Mchare x ‘Calcutta 4’		‘NM275–4’ (Mchare laini × Calcutta 4)	–	–	AA	22
		‘NM258–3’ (Mchare laini × Calcutta 4)	–	–	AA	22
		‘NM209–3’ (Mchare laini × Calcutta 4)	–	–	AA	22
		‘NM237–8’ (Ijihu Inkudu × Calcutta 4)	–	–	AA	22
		‘T.2269–1’ (Huti white × Calcutta 4)	–	–	AA	22
		‘T.2274–6’ (Huti white × Calcutta 4)	–	–	AA	22
		‘T.2274–9’ (Huti white × Calcutta4)	–	–	AA	22
		‘T.2619-15’ (Mchare mlelembo × Calcutta 4)	–	–	AA	22

a Code assigned by the International Transit Centre (ITC, Leuven, Belgium).

The genetic diversity was analyzed using a standardized SSR genotyping platform (Christelová et al., 2011). To achieve higher resolution, the studied set of wild *M. acuminata* accessions and diploid clones was supplemented with selected accessions from our previous studies (Šimoníková et al., 2019, 2020).

Using a set of M13-tailed fluorescent-labeled primers, 19 highly polymorphic SSR loci were amplified, and the allele sizes were measured on the ABI 3730xl DNA analyzer (Applied Biosystems, Foster City, CA, USA), followed by the data analysis using GeneMarker v1.75 (Softgenetics, State College, PA, USA) (Christelová et al., 2011). Dendrograms of selected *M. acuminata* species and cultivated clones were constructed using the Neighbor-Net inference method in the SplitsTree4 program (Huson and Bryant, 2006). Neighbor-Net constructs phylogenetic networks to visualize distance data to show evolutionary relationships and conflict in the data (Bryant and Huson, 2023) by use of the split decomposition method (Bandelt and Dress, 1992a, b).

### Oligo-painting FISH

To characterize chromosome structure within the *Musa* accessions, chromosome-arm-specific painting probes developed by Šimoníková et al. (2019) were used. Individual chromosome arms synthesized as immortal libraries by Arbor Biosciences (Ann Arbor, Michigan, USA) were labeled directly by CY5 fluorochrome or by digoxigenin or biotin, according to Han et al. (2015), with minor modifications: the oligomer libraries were amplified using debubbling PCR according to Immortal Labelling Protocol v2.2. (Daicel Arbor Biocsiences; https://arborbiosci.com/) instead of the emulsion PCR.

Please note that in the reference genome assembly of *M. acuminata* ‘DH Pahang’ (D’Hont et al., 2012), which was originally used to develop banana-specific chromosome-arm painting probes, pseudomolecules 1, 6, and 7 are oriented inversely to the traditional way karyotypes are presented, where the short arms are on the top. The orientation of chromosomes in the present study corresponds with the traditional way karyotypes are presented, as was depicted in our previous study (Šimoníková et al., 2019). The “L” stands for the long arms of chromosomes, and the “S” stands for the short arm of chromosomes in the entire manuscript.

Mitotic metaphase chromosome spreads were prepared according to Šimoníková et al. (2020) from root meristems using the dropping method of protoplast suspension described by Doležel et al. (1998). Fluorescence *in situ* hybridization and image analysis were performed as mentioned previously (Šimoníková et al., 2019). A hybridization mixture containing 50% (v/v) formamide, 10% (w/v) dextran sulfate in 2 × SSC, and 10 ng/µL of labeled probes was added onto a slide and denatured for 90 s at 80°C, followed by overnight hybridization performed in a humid chamber at 37°C. The sites of digoxigenin- and biotin-labeled probes were detected using anti-digoxigenin-FITC (Roche Applied Science, Penzberg, Germany) and streptavidin-Cy3 (ThermoFisher Scientific/Invitrogen, Carlsbad, CA, USA), respectively. The stringent washes, detection of probe signals, and final chromosome counterstaining with DAPI and mounting of the preparations in Vectashield Antifade Mounting Medium (Vector Laboratories, Burlingame, CA, USA) were performed according to Beránková and Hřibová (2023).

### Microscopic and image analysis

The slides were examined with an Axio Imager Z.2 Zeiss microscope (Zeiss, Oberkochen, Germany) equipped with a Cool Cube 1 camera (Metasystems, Altlussheim, Germany) and appropriate optical filters and a PC with ISIS software 5.4.7 (Metasystems, Altlussheim, Germany). The final image adjustment and creation of idiograms were done in Adobe Photoshop CS5. Different probe combinations hybridizing on a minimum of 10 preparations with mitotic metaphase chromosome spreads were used for the final karyotype reconstruction of each genotype.

### Illumina sequencing and data analysis

Genomic DNA was isolated with the NucleoSpin PlantII kit (Macherey-Nagel, Düren, Germany) according to the manufacturer’s recommendations and further sheared by Bioruptor Plus (Diagenode, Liège, Belgium) to achieve an insert size of about 500 bp. Libraries for sequencing were prepared from 2 μg of fragmented DNA using the TruSeq^®^ DNA PCR-free kit (Illumina) and sequenced on a NovaSeq 6000 (Illumina, San Diego, CA, USA), producing 2 × 150-bp paired-end reads to achieve a minimal sequence depth of 25×. Raw data were trimmed for low-quality bases and adapter sequences and to the same length using fastp v.0.20.1 (Chen et al., 2018).

Analysis of the proportion of individual parental subgenomes in the F1 hybrid clones was done using the vcfHunter pipeline (https://github.com/SouthGreenPlatform/vcfHunter), according to Baurens et al. (2019). Briefly, trimmed reads were aligned to reference the genome sequence of *M. acuminata* ssp. *malaccensis* ‘DH Pahang’ v4 (Belser et al., 2021) by BWA-MEM v0.7.15 (Li, 2013), followed by removing redundant reads using MarkDuplicate from Picard Tools v2.7.0, and locally realigned around indels using the IndelRealigner tool of the GATK v3.3 package (McKenna et al., 2010). Bases with a mapping quality of ≥ 10 were counted using the process_reseq_1.0.py python script (https://github.com/SouthGreenPlatform/vcfHunter). Variant calling and SNP filtering steps were performed according to Baurens et al. (2019) using the VcfPreFilter.1.0 python script (alleles supported by at least three reads and with a frequency 0.25 were kept as variant) and the vcfFilter.1.0.py python script (< 6-fold coverage for the minor allele were converted to missing data) (https://github.com/SouthGreenPlatform/vcfHunter). Finally, the proportion of parental genomes in the F1 hybrid clones along the individual chromosomes of the reference genome sequence was called using biallelic SNPs (SNPs specific to Mchare cultivars and *M. acuminata* spp. *burmannicoides* ‘Calcutta 4’) in CDS genome regions using vcf2allPropAndCov.py and vcf2allPropAndCovByChr.py python scripts (https://github.com/SouthGreenPlatform/vcfHunter), according to Baurens et al. (2019).

## Results

To enlarge the knowledge of general chromosome structure in *Musa acuminata* and to shed light on the evolution of *M. acuminata*, we provided cytogenetic analysis in 25 diploid *M. acuminata* accessions, including wild species and natural cultivars. We also analyzed chromosome structure in F1 hybrids obtained from ‘Mchare’ × *M. acuminata* ssp. *burmannicoides* ‘Calcutta 4’ crosses, to shed light on the transfer of Mchare chromosomes differing in their structures in comparison to ‘Calcutta 4’—chromosomes 1, 3 and 8—and corresponding reshuffled chromosome structures: reciprocal translocations between long arms of chromosomes 1 and 4 (1L/4L, 4L/1L) and Robertsonian translocation between chromosome 3 and 8 (3S/8L and 8S/3L).

### Evolutionary relationships within *M. acuminata*

**Figure 1:**
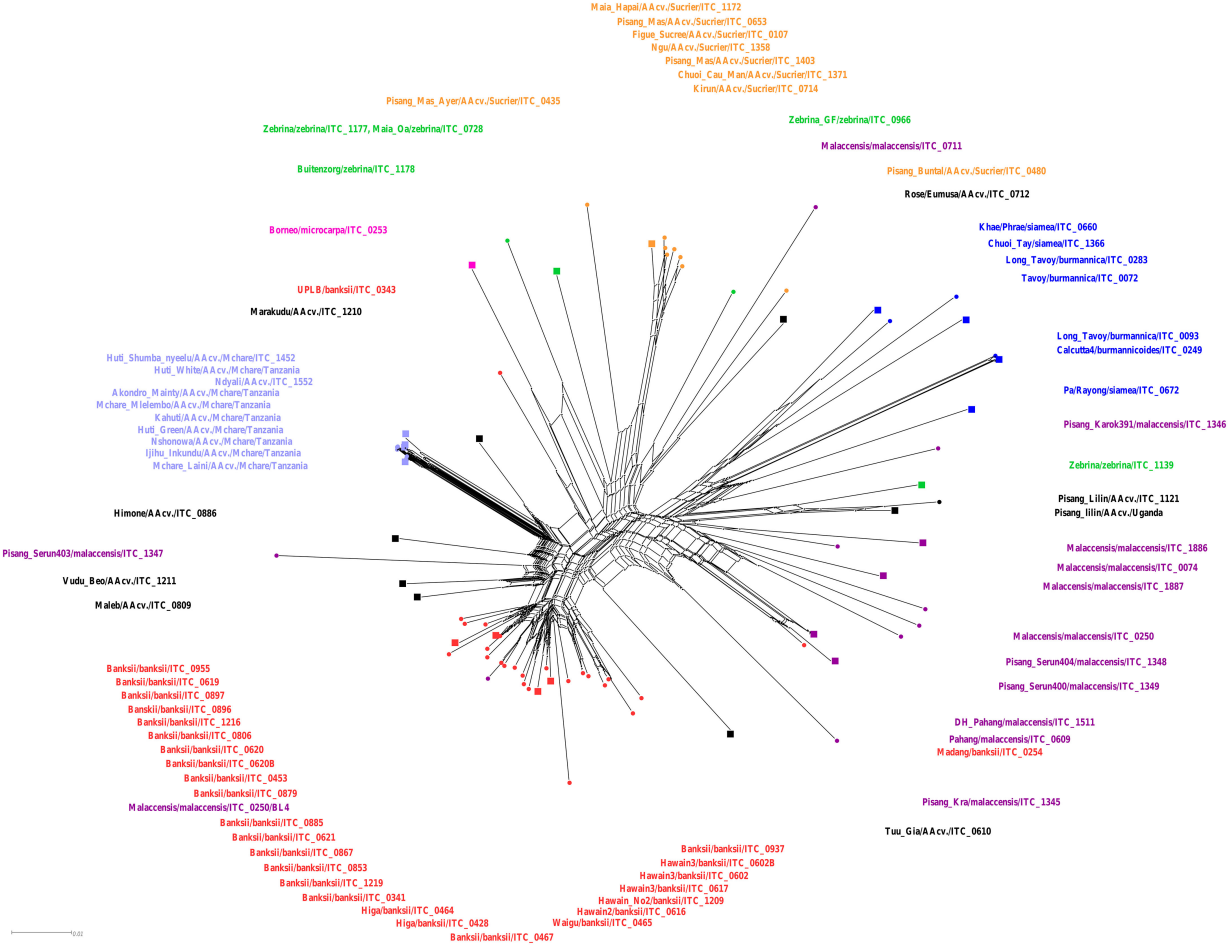
Neighbor-Net analysis of *Musa* accessions performed by SplitsTree. Accessions representing different *M. acuminata* subspecies and groups of cultivars are depicted in colors: *banksii* ssp. in red; *malaccensis* ssp. in violet; *burmannica/burmannicoides/siamea* in blue; *zebrina* in green; *microcarpa* in pink; Mchare genotypes in light blue; Sucrier genotypes in orange; and other analyzed banana AA cultivars in black. The accessions that were used for chromosome painting are depicted as squared nodes in the Neighbor-Net tree.

To assess evolutionary relationships among selected wild species and cultivars of *M. acuminata*, the SSR genotyping data were used to create a phylogenetic network (**Figure 1**). Neighbor-Net of wild *acuminata* subspecies and selected natural diploid hybrids resulted in split-separated populations of ssp. *banksii* from ssp. *burmannica/siamea* group, ssp. *malaccensis* group, and Mchare and Sucrier (Pisang Mas) groups of cultivars. Other analyzed cultivars were clustered in close proximity with the closely related *acuminata* subspecies (**Figure 1**). *M. acuminata* ssp. *microcarpa*, which was represented only by one accession (‘Borneo’), is closely related to *M. acuminata* ssp. *zebrina* (‘Maia Oa’, ‘Zebrina’, and ‘Buitenzorg’ accessions). Some of the accessions did not cluster together with their presumed relatives. For instance, ‘Madang’ cv. (ITC0254) and ‘Zebrina’ ITC1139 clustered together with *malaccensis* accessions; ‘Zebrina GF’ (ITC0966) and ‘Malaccensis’ ITC0711 were included within the Sucrier group. The ‘Malaccensis’ ITC0250/BL4 was most probably a mislabeled sample because it clustered within the *banksii* clade. ‘Pisang Serun’ (*malaccensis*; ITC1347), together with ‘Himone’, ‘Vudu Beo’, and ‘Maleb’ cultivars, shared splits with the Mchare group of accessions, signifying their close relationships (**Figure 1**). Integration of clones representing F1 progeny obtained after Mchare × ‘Calcutta 4’ (ssp. *burmannicoides*) crosses did not change the position and composition of the other group of *acuminata* subspecies (**Supplementary Figure S1**). Most F1 progeny occupied one distinct clade alongside Mchare cultivars. The presence of ‘Calcutta 4’ accession within the F1 hybrids suggests successful hybridization. Two representatives of F1 progeny (NM237–8 and NM237–1) clustered in close proximity to ‘Borneo’, and ‘NM209–14’ F1 hybrid clustered with ‘Rose’, which can indicate that they are not successful hybrids with *M. acuminata* ‘Calcutta 4’, and could be mislabeled (**Supplementary Figure S1**).

### Karyotype structure of *M. acuminata*

**Figure 2:**
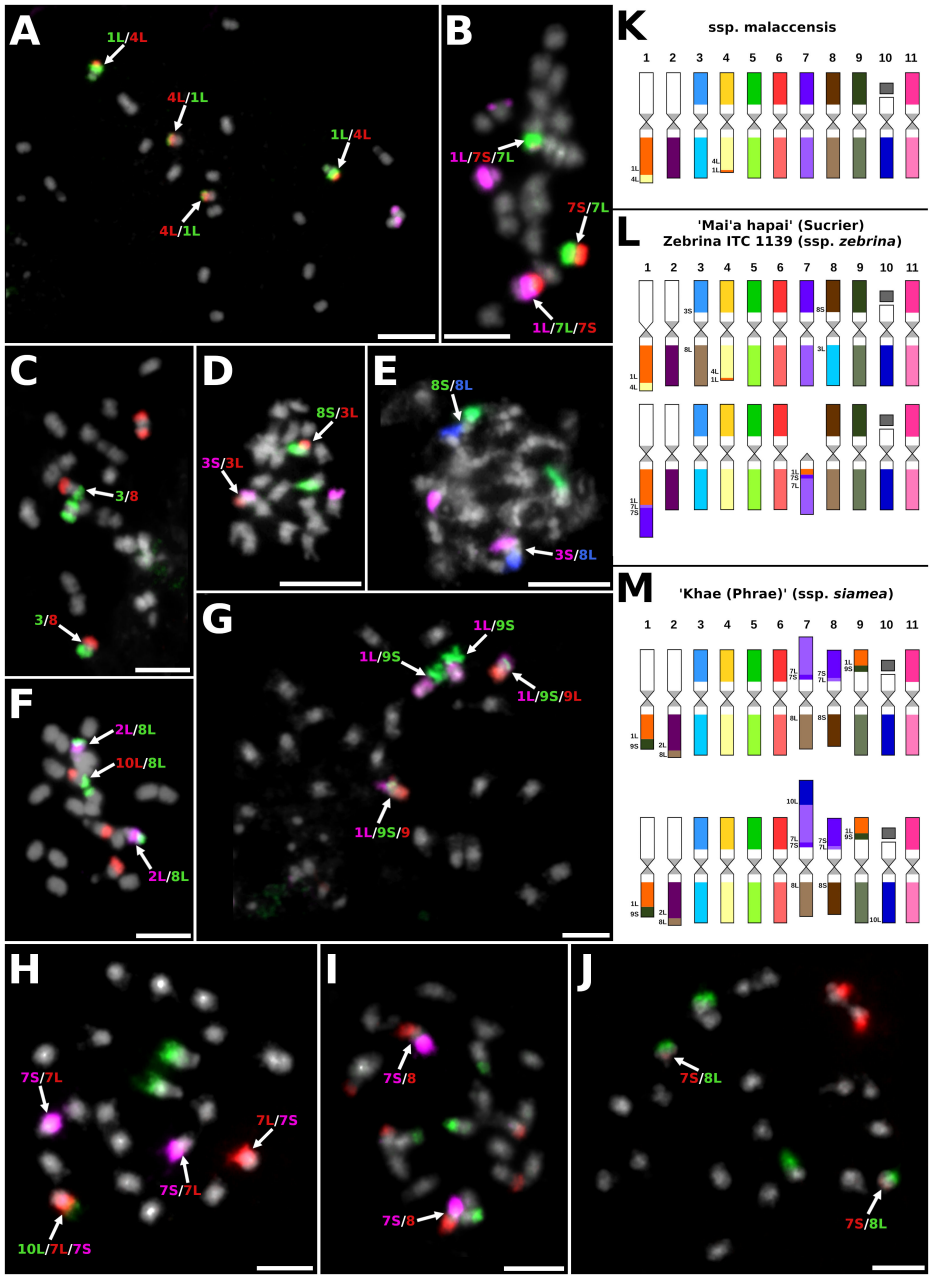
Examples of chromosome translocations identified by oligo-painting FISH on mitotic metaphase plates of *M. acuminata* subspecies and their natural hybrids: **(A)** ITC1886 *M. acuminata* ssp. *malaccensis* (2*n* = 2*x* = 22), probes for long arm of chromosome 1, entire chromosome 4, and short arm of chromosome 9 labeled in green, red, and purple, respectively. **(B)** ITC1172 ‘Mai’a hapai’ (AA, subgr. Sucrier, 2*n* = 2*x* = 22), probes for long arm of chromosome 1, short arm of chromosome 4, and long arm of chromosome 4 were labeled in purple, red, and green, respectively. **(C)** ITC1172 ‘Mai’a hapai’, probes for chromosomes 3 and 8 were labeled in green and red, respectively. **(D)** ITC1172 ‘Mai’a hapai’, probes for short arm of chromosome 3, long arm of chromosome 3, and short arm of chromosome 8 were labeled in purple, red, and green, respectively. **(E)** ITC1172 ‘Mai’a hapai’, probes for short arm of chromosome 3, short arm of chromosome 8, and long arm of chromosome 8 were labeled in purple, green, and blue pseudocolor. **(F)** ITC0660 ‘Khae (Phrae)’ (ssp. *siamea*, 2*n* = 2*x* = 22), probes for long arm of chromosome 2, long arm of chromosome 8, and long arm of chromosome 10 were labeled in purple, green, and red, respectively. **(G)** ITC0660 ‘Khae (Phrae)’, probes for long arm of chromosome 1, short arm of chromosome 9, and long arm of chromosome 9 were labeled in purple, green, and red, respectively. **(H)** ITC0660 ‘Khae (Phrae)’, probes for long arm of chromosome 10, short arm of chromosome 7, and long arm of chromosome 7 were labeled in green, purple, and red, respectively. **(I)** ITC0660 ‘Khae (Phrae)’, probes for short arm of chromosome 7, short arm of chromosome 8, and long arm of chromosome 9 were labeled in purple, red, and green, respectively. **(J)** ITC0660 ‘Khae (Phrae)’, probes for short arm of chromosome 7 and long arm of chromosome 8 were labeled in red and green, respectively. Chromosomes were counterstained with DAPI (light grey pseudocolor). Arrows point to chromosomes with translocations. Bars = 5 µm. Idiograms of four *Musa acuminata* representatives (2*n* = 2*x* = 22): **(K)** *M. acuminata* ssp. *malaccensis* (ITC1886 and ITC1887); **(L)** *M. acuminata* ssp. *siamea* ‘Khae (Phrae)’ (ITC0660); and **(M)** clone ‘Mai’a hapai’ (Sucrier; ITC1172). Chromosomes are oriented with their short arms on the top and long arms on the bottom in all idiograms, and translocated parts of the chromosomes contain extra labels.

To perform comparative karyotyping and reveal chromosome structural changes within *M. acuminata* accessions, oligo-painting FISH was used (**Figures 2A–M**; **Supplementary Figure S2**). The painting probes were originally designed for the reference genome sequence of *M. acuminata* ssp. *malaccensis* ‘DH Pahang’ (D’Hont et al., 2012). Therefore, all chromosome structural rearrangements mentioned in the study are described based on comparison to the standard chromosome set of the ‘DH Pahang’ reference genome sequence, according to Šimoníková et al. (2019).

The chromosome painting of two *malaccensis* accessions (ITC1886 and ITC1887) revealed the presence of reciprocal translocation of short segments of the long arms of chromosomes 4 and 1, which was present in the homozygous state (**Figure 2A**). The same type of translocation was also revealed in a heterozygous state in the edible cultivars ‘Vudu Beo’, cv. ‘Rose’ and ‘Mai’a hapai’ (**Supplementary Figures S2A–C**). Cultivar ‘Rose’ contained additional chromosomal structural changes, which were observed in one chromosome set. Translocation of the short arm of chromosome 7 to the long arm of chromosome 1, which resulted in the formation of a small telocentric chromosome consisting of only 7L (**Supplementary Figure S2B**). Cultivar ‘Mai’a hapai’, which is a representative of Sucrier subgroup, also contained additional large rearrangements between chromosomes 1L and 7, again in the heterozygous state. This rearrangement led to the formation of a recombined chromosome containing the short segment of 7L and short arm of 7S translocated to 1L and a telocentric chromosome containing short segment of chromosome 1L and 7S and the long arm of chromosome 7 (**Figure 2B**; **Supplementary Figure S2C**). In addition, cultivar ‘Mai’a hapai’ contained Roberstonian translocation between chromosomes 3 and 8 in the homozygous state (**Figures 2C–E**). Interestingly, one genotype is described as ssp. *zebrina* (Zebrina, ITC1139) had the same chromosome structure as the cultivar ‘Mai’a hapai’ (**Supplementary Figure S2C**).

The karyotype analysis of *M. acuminata* ssp. *siamea* ‘Khae (Phrae)’ showed the presence of translocations between chromosome arms 1L and 9S and 2L and 8L (**Figures 2F, G**), which were described previously in closely related accessions of *burmannica*/*burmannicoides*/*siamea* (Šimoníková et al., 2019). The genome of ‘Khae (Phrae)’ contained additional translocations, which included chromosomes 7 and 8. A recombined chromosome containing a short arm of chromosome 7, a short segment of 7L, and a short arm of chromosome 8 was found in the homozygous state (**Figures 2I, J**; **Supplementary Figure S2D**). The reciprocal recombined chromosome, containing a short arm of chromosome 7, a short segment of 7L, and the long arm of chromosome 8 was found in the heterozygous state, as was the recombined chromosome, consisting of a segment of chromosome 10L, a long arm of chromosome 7, a short segment of 7S, and a long arm of chromosome 8 (**Figure 2H**; **Supplementary Figure S2D**).

No chromosome rearrangement was detected in the two representatives of the *banksii* subspecies (ITC0341 and ITC0896) as compared to the ‘DH Pahang’ reference banana genome (**Supplementary Figure S2E**). On the other hand, a small segment of the short arm of chromosome 9 was inserted into the long arm of chromosome 5 near the centromeric region in another *banksii* representative, ‘Higa’ (**Supplementary Figure S2F**). No chromosome rearrangements were observed in the edible cultivar ‘Marakudu’ (**Supplementary Figure S2E**). Edible banana cultivars ‘Himone’ and ‘Maleb’ share the same chromosome structures, containing one reciprocal translocation between the short arm of chromosome 3 and the long arm of chromosome 8, in a heterozygous state (**Supplementary Figure S2G**), which was previously identified in *zebrina* subspecies (Šimoníková et al., 2020). Cultivar ‘Tuu Gia’ comprised reciprocal translocation between chromosomes 1L and 9S and translocation between 2L and 8L, which were also present in *burmannica*/*burmannicoides*/*siamea* accessions (**Supplementary Figure S2H**; Šimoníková et al., 2020). Another chromosome rearrangement involved reciprocal translocation between chromosomes 7 and 8, which led to the formation of chromosome structures consisting of the long arm of chromosome 7, a small segment of chromosome 8S, and the long arm of chromosome 8, and to a recombined chromosome consisting of the short arm of chromosome 7 and a large segment of the short arm of chromosome 8. All of these translocations were observed in a heterozygous state (**Supplementary Figure S2H**).

The three Mchare banana cultivars analyzed in our present study (Mchare mlelembo, Mchare Laini, and Kahuti) had the same genome structure as previously analyzed cultivars belonging to the Mchare group (Šimoníková et al., 2020). All Mchare genotypes contained two reciprocal translocations involving chromosomes 4 and 1 and chromosomes 3 and 8. Both reciprocal translocations were found in the heterozygous state (**Supplementary Figure S2I**). All chromosome changes (translocations) observed in our present study are listed in **Supplementary Table S1**.

### Karyotype structure of Mchare × ‘Calcutta 4’ progeny

**Figure 3:**
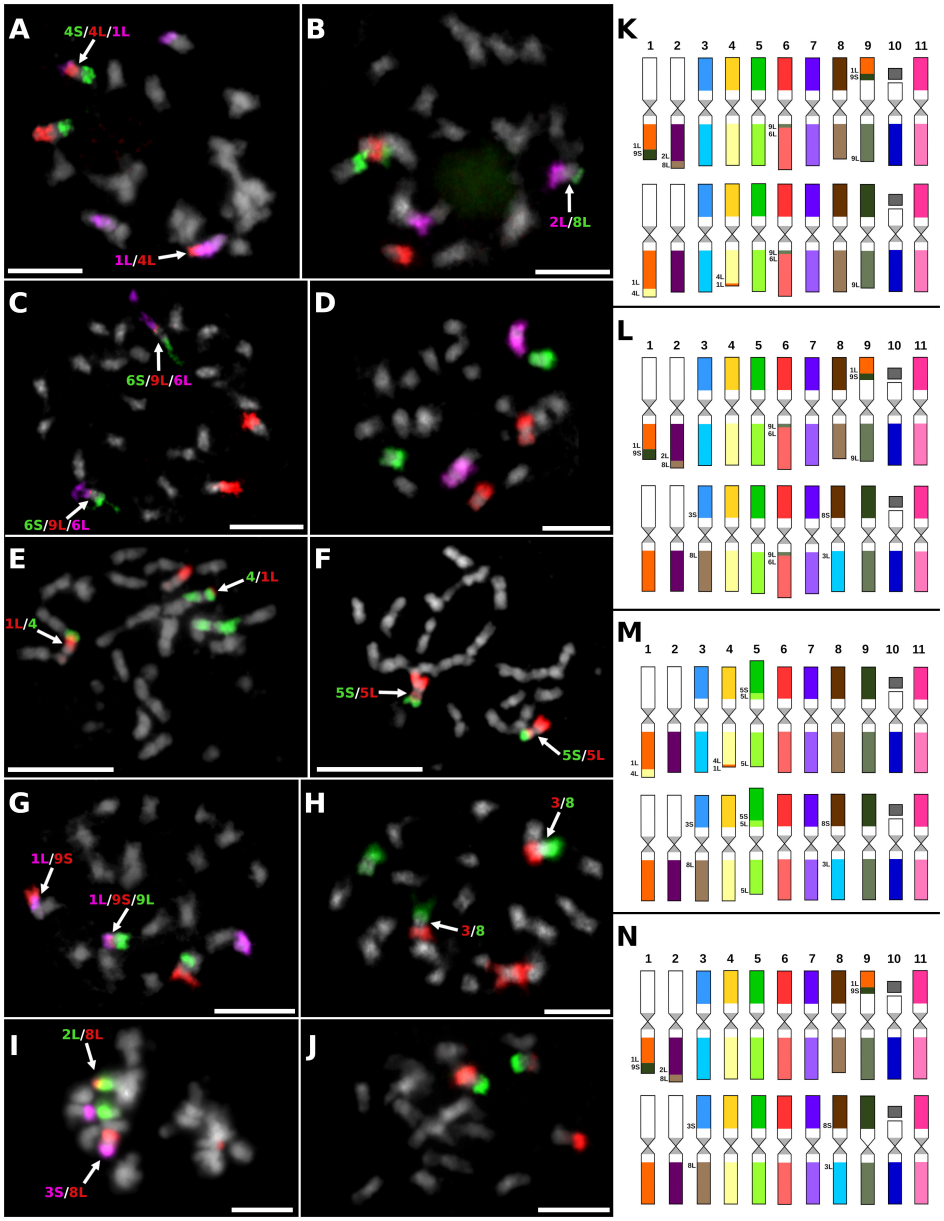
Examples of chromosome translocations identified by oligo-painting FISH on mitotic metaphase plates of seven F1 hybrids obtained in improvement programs of Mchare: **(A)** ‘NM275–4’ (2*n* = 2*x* = 22), probes for long arm of chromosome 1, short arm of chromosome 4, and long arm of chromosome 4 were labeled in purple, green, and red, respectively. **(B)** ‘NM258–3’ (2*n* = 2*x* = 22), probes for long arm of chromosome 2, short arm of chromosome 8, and long arm of chromosome 8 were labeled in purple, red, and green, respectively. **(C)** ‘NM253–3’ (2*n* = 2*x* = 22), probes for short arm of chromosome 6, long arm of chromosome 6, and long arm of chromosome 9 were labeled in green, purple, and red, respectively. **(D)** ‘NM209–3’ (2*n* = 2*x* = 22), probes for chromosomes 5, 6, and 7 were labeled by red, purple, and green, respectively. **(E)** ‘NM237–8’ (2*n* = 2*x* = 22), probes for long arm of chromosome 1 and entire chromosome 4 were labeled in red and green, respectively. **(F)** ‘NM237–8’ (2*n* = 2*x* = 22), probes for short arm of chromosome 5 and long arm of chromosome 5 were labeled in red and green, respectively. **(G)** ‘T.2274–6’ (2*n* = 2*x* = 22), probes for long arm of chromosome 1, short arm of chromosome 9, and long arm of chromosome 9 were labeled in purple, red, and green, respectively. **(H)** ‘T.2274–6’ (2*n* = 2*x* = 22), probes for chromosomes 3 and 8 were labeled in red and green, respectively. **(I)** ‘T.2774–9’ (2*n* = 2*x* = 22), probes for long arm of chromosome 2, short arm of chromosome 3, and long arm of chromosome 8 were labeled in green, purple, and red, respectively. **(J)** ‘T.2619–15’ (2*n* = 2*x* = 22), probes for short arm of chromosome 9 and long arm of chromosome 9 were labeled in green and red, respectively. Chromosomes were counterstained with DAPI (light grey pseudocolor). Arrows point to translocation chromosomes. Bars = 5 µm. Idiograms of F1 hybrid clones that originated from crosses between Mchare cultivars and *M. acuminata* ssp. *burmannicoides* ‘Calcutta 4’ (2*n* = 2*x* = 22): **(K)** clone ‘NM275–4’; **(L)** clone ‘NM258–3’; **(M)** clone ‘NM237–8’; and **(N)** clones ‘T.2274–6’, ‘T.2619–9’, ‘T.2774-15’, and ‘NM209–3’. Chromosomes are oriented with their short arms on the top and long arms on the bottom in all idiograms, and translocated parts of the chromosomes contain extra labels.

The chromosome painting was also used to study the genome structure of eight F1 hybrids, which were obtained by crosses between different Mchare genotypes (female parent) and the wild species *M. acuminata* ssp. *burmannicoides* ‘Calcutta 4’ (male parent) (**Figures 3A–N**; **Supplementary Figure S2J**). As it was revealed previously (Šimoníková et al., 2020), the genome of *M. acuminata* ssp. *burmannicoides* ‘Calcutta 4’ contains a specific chromosome structure for two pairs of chromosomes that differ from the chromosome structure found in Mchare genotypes. Based on this information, the F1 progeny of crosses between Mchare and ‘Calcutta 4’ should contain one chromosome set inherited from the Mchare cultivar and one chromosome set inherited from ‘Calcutta 4’. Thus, the hypothetical genome composition of such F1 hybrid clones can be represented by 16 different combinations of chromosomes varying in their structure between the parental genotypes (**Supplementary Figure S3**).

The genome of seven F1 hybrid clones contained one set of translocation chromosomes specific to ‘Calcutta 4’, and any of these seven F1 hybrid clones contained both sets of translocation chromosomes specific to Mchare (**Figures 3A–D, G–N**; **Supplementary Figure S2J**). The presence of any translocated chromosome specific to ‘Calcutta 4’ was not observed after chromosome painting in one F1 hybrid clone ‘NM237–8’ (**Figures 3E, F**). Oligo-painting FISH of this F1 hybrid clone, which had arisen from a cross between ‘Ijihu Inkudu’ (Mchare type) and ‘Calcutta 4’, resulted in the same genome structure as Mchare clones (**Figure 3M**; **Supplementary Figure S2I**). One F1 hybrid clone (‘NM275–4’) inherited 1L/4L and 4L/1L translocation chromosomes from the Mchare genome (**Figures 3A, K**), and five of the remaining F1 hybrids (‘NM258–3’, ‘NM209–3’, ‘T.2274–6’, T.2274–9’, and ‘T.2274–15’) inherited 3S/8L and 8S/3L recombined chromosomes from the Mchare genome (**Figures 3H, I, L, N**). A short segment of chromosome 9L was inserted into the long arm of chromosome 6 near the centromeric region in ‘NM275–4’ and ‘NM258–3’ F1 hybrid clones (**Figures 3C, K, L**). None of the recombined chromosomes specific to Mchare was transmitted to the F1 hybrid clone ‘T.2269–1’ (**Supplementary Figure S2J**).

### Genome constitution of Mchare × ‘Calcutta 4’ F1 hybrids

**Figure 4:**
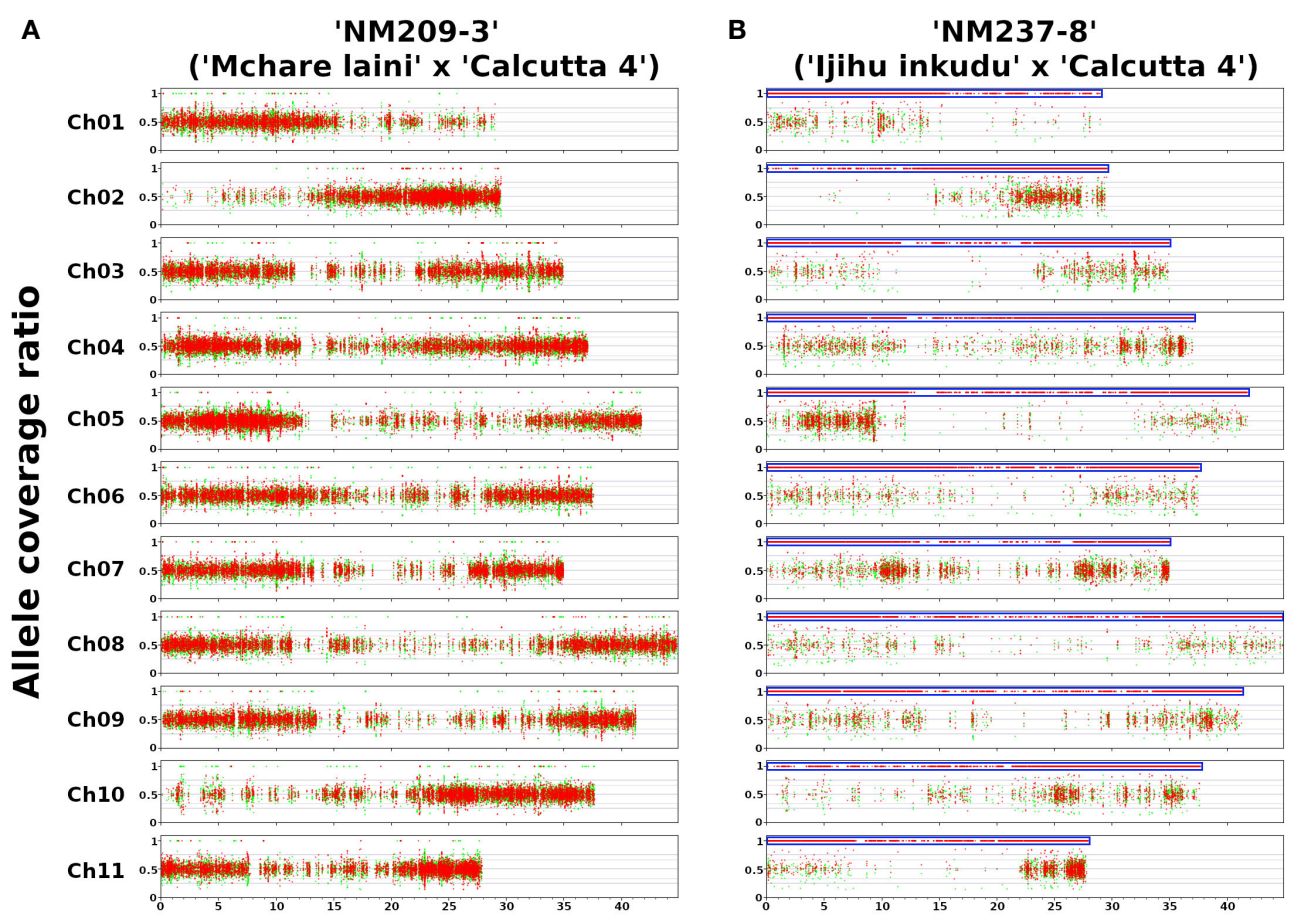
Genome structure of F1 hybrid clones gained after crosses of Mchare banana cultivars (female parent) and *M. acuminata* ssp. *burmannicoides* ‘Calcutta 4’ (male parent). The *y*-axis represents the coverage ratio of alleles specific to Mchare genotypes (red dots) and to the *M. acuminata* ssp. *burmannicoides* ‘Calcutta 4’ (green dots) along 11 chromosomes of *M. acuminata* ssp*. malaccensis* ‘DH Pahang’ reference genome sequence (*x*-axis). An allele coverage ratio (ACR) of 0.5 represents the equal contribution of the two parental genomes, and an ACR of 1.0 depicts the exclusive representation of one parental genome. **(A)** The expected ratio of 50%:50% of ‘Mchare’ versus ‘Calcutta 4’-specific SNPs (ACR = 0.5) was revealed in most F1 hybrid clones, including ‘NM209–3’. **(B)** A ratio of 100%:0% of ‘Mchare’ versus ‘Calcutta 4’-specific SNPs (ACR = 1.0) was revealed only in one F1 hybrid clone ‘NM237–8’. The blue boxes indicate chromosome regions with exclusive representation of ‘Mchare’-specific SNPs in the F1 hybrid clone ‘NM237–8’. The detection of the ‘Calcutta 4’-specific SNPs along the chromosome arms of reference genome sequence *M. acuminata* ‘DH Pahang’ was most probably caused by the fact that the reference genome sequence used for reads alignment was created from the more distinct subspecies *malaccensis*. A small proportion of alleles specific to *burmannica*/*burmannicoides* subspecies were present also in the Chicame cultivar of Mchare bananas from Comoros, as shown in Martin et al. (2020a).

To further complete information on the genome composition of analyzed F1 hybrid clones, we performed Illumina resequencing of the F1 hybrids and their parental genomes. The proportion of parental genomes in the F1 progeny was identified by the VcfHunter program pipeline (Baurens et al., 2019). SNPs specific to parental genomes were depicted based on the alignment of Illumina reads to reference the genome sequence of *M. acuminata* ssp. *malaccensis* ‘DH Pahang’. The coverage ratio of parental-specific SNPs along 11 chromosomes of the reference genome sequence (**Supplementary Table S2**) showed that most F1 hybrid clones contained whole haploid sets of chromosomes representing the individual parents (**Figure 4A**; **Supplementary Figure S4**). The only exception was revealed for the clone ‘NM237–8’, which contained only SNPs specific to Mchare (**Figure 4B**) and any chromosome region specific to *M. acuminata* ‘Calcutta 4’ was not revealed. This observation corresponds with the results of chromosome painting, which also did not detect any chromosome carrying ‘Calcutta 4’-specific chromosome structure (**Figures 2C, J**).

## Discussion

Complex phylogenetic relationships within *Musa* and edible banana cultivars shown by Neighbor-Net analysis correspond to previous studies (e.g., Perrier et al., 2011; Janssens et al., 2016; Christelová et al., 2017; Sardos et al., 2022; Martin et al., 2023a). Some of the analyzed accessions, mostly those with hybrid origins, which were further confirmed by oligo-painting, showed unexpected/conflicting positions in the Neighbor-Net graph. The high level of admixture and mosaic genome structure of cultivated banana clones originating from hybridizations between subspecies of *M. acuminata* was proposed recently by Martin et al. (2020a; 2023a, b). Reticulate evolution, caused by polyploidy and hybridization (i.e., allopolyploidy), can promote rapid diversification of numerous plant lineages (reviewed in Stull et al., 2023).

Ancient polyploidization and hybridization events are known to be the major drivers of plant diversification and speciation. These processes lead to the multiplication of chromosome sets in genomes or “genome upsizing” (Ibarra-Laclette et al., 2013; Wendel, 2000), followed by the postpolyploid diploidization processes, which are thought to be associated with extensive loss of DNA or “genome downsizing”, and structural chromosomal changes (Schubert and Vu, 2016; Mandáková et al., 2017; Wang et al., 2021; Farhat et al., 2023). Until now, this phenomenon has been studied in detail mainly in the *Poaceae* and *Brassicaceae* families (e.g., Salse, 2016; Mandáková et al., 2017; Mandáková and Lysak, 2018). Recent developments in oligo-painting FISH and long sequencing technologies that enable the production of chromosome-scale genome sequences even in nonmodel species permit the study of this phenomenon in other plant species.

In the current study, we analyzed and compared the chromosome structure in *M. acuminata* and their natural and artificial hybrids by chromosome-arm-specific oligo-painting FISH, which facilitates revealing large chromosome translocations (Šimoníková et al., 2019, 2020). Genetic linkage between different chromosome-specific markers suggested the presence of five different reciprocal chromosome translocations (between chromosomes: 1 and 4; 2 and 8; 1 and 9; 1 and 7; and 3 and 8) in *M. acuminata* subspecies and their hybrids (Martin et al., 2017; Dupouy et al., 2019; Martin et al., 2020b). These and additional translocations were detected by chromosome-arm-specific oligo-painting FISH (Šimoníková et al., 2019, 2020). Eight additional chromosome rearrangements in *M. acuminata* were revealed in our present work (**Supplementary Table S1**).

Even though the chromosome painting of the additional three *banksii* genotypes did not reveal any presence of large chromosomal translocations, we detected a short segment of chromosome 9S inserted into the peri-centromeric region of chromosome 5 in the ‘Higa’ accession (**Supplementary Figure S2F**). Unfortunately, based on the oligo-painting method, we are not able to distinguish whether this short segment represents duplication or a translocation event.

Despite the very close evolutionary relationships of *burmannica*, *burmannicoides*, and *siamea* subspecies (e.g., Perrier et al., 2009; Christelová et al., 2017; Dupouy et al., 2019), their representatives contained a large number of variable chromosome structures in their genomes. All species contained already described large translocations between chromosomes 1 and 9, and 2 and 8, which seem to be specific for this evolutionary group (Šimoníková et al., 2020; Martin et al., 2020b). On the other hand, using chromosome painting, additional translocations in the genomes of *burmannica–burmannicoides*–*siamea* representatives were revealed. Šimoníková et al. (2020) described the presence of translocations between chromosomes 3 and 4 in ‘Pa Rayong’ (ssp. *siamea*), and reciprocal translocations between chromosomes 7 and 8 and 3 and 8 in the genome of ‘Tavoy’ (ssp. *burmannica*), all presented in the homozygous state. The presence of reciprocal translocation between chromosomes 7 and 8 in the homozygous state was suggested based on genetic linkage patterns in ‘Khae Phrae’ and confirmed cytogenetically by BAC-FISH in ‘Long Tavoy’ in the work of Martin et al. (2020b). The translocation breakpoints were located between 21.8 and 26.3 Mb of ‘Pahang’ reference chromosome 7 and between 22.6 and 32.1 Mb of ‘Pahang’ reference chromosome 8 (Martin et al., 2020b). In comparison, chromosome oligo-painting of ‘Khae Phrae’ performed in our current work revealed the presence of specific chromosome structures containing chromosomes 7, 8, and 10 (**Figure 2M**; **Supplementary Figure 2D**). These reciprocally recombined chromosomes, which contained a short arm of chromosome 7, a short segment of 7L, the long arm of chromosome 8, a segment of chromosome 10L, the long arm of chromosome 7, a short segment of 7S, and the long arm of chromosome 8, were found in the heterozygous state (**Figures 2H–J**; **Supplementary Figure 2D**). Unfortunately, the chromosome oligo-painting method did not enable to localize translocation breakpoints, so we were not able to find out if the short segments of chromosomes 7L or 7S in the chromosome structures mentioned above are consequences of translocation or duplication events. Additional work utilizing long-read sequencing technologies and further whole genome assembly have to be used to unambiguously answer these questions.

In our current study, the *burmannica–burmannicoides*–*siamea* phylogenetic clade contained also one accession (Pisang Karok391) originally described as representative of ssp. *malaccensis*, which thus seems to be mislabeled. On the other hand, the *malaccensis* group of subspecies with the inclusion of ‘Pisang lilin’ and accession described as Zebrina (*M. acuminata* ssp. *zebrina*) represents a sister cluster to *burmannica–burmannicoides*–*siamea*, indicating their common evolutionary history. As it was mentioned earlier, the polyploidization and hybridization events, which were accompanied by large chromosomal rearrangements (Schubert and Vu, 2016; Mandáková et al., 2017; Wang et al., 2021; Farhat et al., 2023), are known to be the major drivers of plant diversification and speciation. We can therefore speculate that the presence of specific translocations events in the *burmannica–burmannicoides*–*siamea* group of subspecies might have had a direct effect on their own diversification and separation from the *malaccensis* subspecies (Janssens et al., 2016). The close relationship between *malaccensis* accessions and the Pisang lilin cultivar is supported by the presence of reciprocal translocation between chromosomes 1 and 4 in their genomes.

Phylogenetic analysis showed that the only representative of ssp. *microcarpa* ‘Borneo’ is closely related to geographically close accession ‘Buitenzorg’ (described as *zebrina* ssp.) and one accession described as ‘Pisang Mas Ayer’ (Sucrier), a sister group of the other two accessions of the *zebrina* subspecies (**Figure 1**; **Supplementary Figure 1**). Unfortunately, ‘Buitenzorg’ and ‘Pisang Mas Ayer’ were not available during our study for the oligo-painting FISH to analyze their chromosome structure. We can thus only speculate that these two accessions can be of complex/hybrid origin, explaining their unexpected/conflicting position in the Neighbor-Net graph. This speculation can also be supported by previous results of chromosome painting that did not reveal the presence of any specific translocation event in the genome of the ‘Borneo’ accession, which could indicate a close relationship to the *zebrina* subspecies or Sucrier cultivars that were analyzed so far (Šimoníková et al., 2020). High level of molecular heterogeneity in ssp. *microcarpa* was mentioned in the study of Perrier et al. (2009). Previous studies showed that ‘Borneo’ accession shared alleles with *banksii* and always clustered together with *banksii* accessions (Perrier et al., 2009; Martin et al., 2023a). Perrier et al. (2009) showed that other representatives of *microcarpa* were closer to *zebrina*. Unfortunately, in all the studies mentioned, only a small number (at the most two representatives) of *microcarpa* accessions were analyzed; thus, we cannot speculate about the speciation processes that led to the origin and diversification of this subspecies. Nevertheless, the high level of molecular heterogeneity mentioned by Perrier et al. (2009) can indicate that hybridization events could play a major role in the speciation of the *microcarpa* subspecies. To shed more light on the evolution and genome structure of ssp. *microcarpa*, a larger number of *microcarpa* accessions from different geographic areas have to be collected and analyzed.

The z*ebrina* genotypes did not form a distinct phylogenetic clade, and most of them clustered in close proximity to Sucrier cultivars. The genomes of ssp. *zebrina* and Sucrier cultivars contain reciprocal Robertsonian translocation between chromosomes 3 and 8 in the homozygous and heterozygous state, respectively. The chromosome painting showed that the Sucrier cultivar also contained additional translocation events, all in the heterozygous state. Reciprocal translocation between chromosomes 1 and 4, specific to the *malaccensis* group, and translocation between chromosomes 1 and 7 (**Figures 2B, L**; **Supplementary Figure S2C**) indicate the involvement of a geographically close subspecies *zebrina* and *malaccensis* in the origin of Sucrier cultivars. Studies by Martin et al. (2020a, 2023a) indicated that at least four different progenitors were involved in the origin of Sucrier cultivars—*malaccensis*, *zebrina*, *banksii*, and an unknown M_2 progenitor. The same results of chromosome painting were obtained for Zebrina ITC1139. Even though the genomes of Zebrina ITC1139 and Sucrier cultivar ‘Ma’i hapai’ shared the chromosome painting structures, an unexpected phylogenetic position of ‘Zebrina’ ITC1139 was found. ‘Zebrina’ ITC1139 clustered together with ‘Pisang lilin’. This result can indicate the hybrid origin of the ‘Zebrina’ ITC1139, whose genome can arise by several rounds of hybridization, similar to Sucrier, but contains a large proportion of *malaccensis*-like genome regions.

The genomic constitution of edible banana cultivars showed a high level of admixture (Martin et al., 2020a; Martin et al., 2023a). The Robertsonian translocation between chromosomes 3 and 8 was also observed in the genomes of two additional banana hybrid clones, ‘Himone’ and ‘Maleb’. Neighbor-Net inference showed that these clones, together with the other two cultivars, ‘Vudu Beo’ and ‘Marakudu’, are closely related to Mchare. Similarly to other studies (Christelová et al., 2017; Perrier et al., 2019; Martin et al., 2020a), Mchare representatives formed a distinct evolutionary clade, which indicates their exceptional position in banana evolution. Previous phylogenetic studies were not able to unambiguously identify the mode of evolution and origin of this important group of edible banana clones, which represents a unique genetic source within *Musa*. It was shown that two subspecies of *M. acuminata*, ssp. *zebrina* and *banksii* shared some alleles with the Mchare clones (Perrier et al., 2009; Hippolyte et al., 2012; Perrier et al., 2019). The involvement of *zebrina* in the origin of Mchare bananas is supported by oligo-painting FISH, which confirmed the presence of a Robertsonian translocation between chromosomes 3 and 8 in the genomes of all Mchare clones analyzed in the present study and previously by Šimoníková et al. (2020). On the other hand, no Mchare banana cultivar was found in the region, where *banksii* and *zebrina* subspecies occur and could have contributed to the origin of these cultivars (Perrier et al., 2009; Hippolyte et al., 2012).

Chromosome painting was used to analyze the genome structure of several F1 Mchare hybrids resulting from different crosses between Mchare and *M. acuminata* ssp*. burmannicoides* ‘Calcutta 4’. Regarding the fact that oligo-painting FISH can only reveal those chromosomes consisting of specific structures (the presence of translocations), it can only provide partial information on the whole genome composition of the F1 progenies. As expected for intersubspecific crosses, all progenies, except NM237–8, contained one chromosome set from both parents. This was confirmed by Illumina resequencing, which identified the contribution of parental subgenomes to the hybrid clones. The identification of parental-specific SNPs and their distribution along the chromosomes of reference genome sequence *M. acuminata* ‘DH Pahang’ revealed a balanced presence of both parental genomes in all F1 hybrids. The only exception was the clone NM237–8, which contained only Mchare-specific SNPs, suggesting that the clone might have originated from an unreduced Mchare gamete. Genome composition analysis using Illumina resequencing did not reveal the presence of any aneuploid genome regions in F1 hybrids.

This analysis was done with the reference genome sequence of *M. acuminata* ssp. *malaccensis* (version 4), which is distinct from Mchare and ‘Calcutta 4’. Therefore, we cannot exclude the presence of genome regions that do not contain a proportional representation of parental subgenomes. Future high-quality chromosome-scale assembly of Mchare and/or other parental genomes used in the crosses will provide detailed information on the genome composition of the hybrid progenies.

## Data availability statement

Illumina sequencing data are available on NCBI sequence Read Archive (SRA) under the BioProject ID: 1033816 (SRA experiments: SRX22339926 - SRX22339938). The SNP files which were used for in silico painting of F1 hybrid clones are available in the Dryad repository (https://doi.org/doi:10.5061/dryad.44j0zpcnq).

## Author contributions

DB: Investigation, Methodology, Project administration, Validation, Writing – original draft, Writing – review & editing. JČ: Investigation, Validation, Visualization, Writing – review & editing. GM: Formal analysis, Investigation, Software, Writing – review & editing. AD: Investigation, Writing – review & editing. HM: Resources, Writing – review & editing. AB: Resources, Writing – review & editing. RS: Resources, Writing – review & editing. EH: Conceptualization, Data curation, Formal analysis, Funding acquisition, Investigation, Visualization, Writing – original draft, Writing – review & editing.

## Supplementary material

Supplementary Figure 1 Neighbor-Net analysis of *Musa* accessions and F1 hybrids between Mchare and *M. acuminata* ssp. *burmannicoides* ‘Calcutta 4’ performed by SplitsTree. Accessions representing different *M. acuminata* subspecies and group of cultivars are depicted in colors: *banksii* ssp. in red; *malaccensis* ssp. in violet; *burmannica/burmannicoides/siamea* in blue; *zebrina* in green; *microcarpa* in pink; Mchare genotypes in light blue; Sucrier genotypes in orange; other analyzed banana AA cultivars in black; and F1 progeny of Mchare and *M. acuminata* ssp. *burmannicoides* ‘Calcutta 4’ in brown. The accessions which were used for chromosome painting are depicted as squared nodes in the neighbor net tree.

Supplementary Figure 2 Idiograms and short translocations (duplications) observed by oligo painting FISH in genomes of analyzed *M. acuminata* species and its edible banana clones. **(A)** Idiogram of cv. ‘Vudu Beo’ ITC1211; **(B)** Idiogram of cv. ‘Rose’ ITC0712 and oligo painting FISH with the probes for long arms of chromosomes 1 and 4, and oligo painting FISH with the probes for long arm of chromosome 1, short arm of chromosome 7 and long arm of chromosome 7; **(C)** Idiogram of *M. acuminata* ssp. *zebrina* ITC1139 and cv. ‘Mai’a hapai’ ITC1172, and oligo painting FISH with the probes for long arm of chromosome 1, and probes for short and long arms of chromosome 7; **(D)** Idiogram of *M. acuminata* ssp. *siamea* ‘Khae (Phrae)’ ITC0660, and oligo painting FISH with the probes for short arm of chromosome 7, long arm of chromosome 7 and long arm of chromosome 10L, and oligo painting FISH with the probes for short arm of chromosome 7 and probes for entire chromosomes 8 and 9; **(E)** Idiogram of *M. acuminata* ssp. *banksii* ITC0341 and ITC 0896, and cv. ‘Marakudu’ ITC1210; **(F)** Idiogram of *M. acuminata* ssp. *banksii* ‘Higa’ ITC0428 and oligo painting FISH with the probes for short arm of chromosome 9, short arm of chromosome 5 and long arm of chromosome 5; **(G)** Idiogram of cv. ‘Himone’ ITC0886 and cv. ‘Maleb’ ITC0809; **(H)** Idiogram of cv. ‘Tuu Gia’ ITC0610 and oligo painting FISH with the probes for long arm of chromosome 1 and entire chromosome, oligo painting FISH with the probes for long arm of chromosome 2 and entire chromosome 8, and oligo painting FISH with the probes long arms of chromosomes 7 and 8; **(I)** Idiogram of cv. ‘Mchare mlelembo’, cv. ‘Mchare laini’ and cv. ‘Kahuti’; and idiogram of F1 hybrid clone gained after crosses of Mchare banana cultivars (female parent) and *M. acuminata* ssp. *burmannicoides* ‘Calcutta 4’ (male parent): **(J)** ‘T2269–1’ (2n=2x=22).

Supplementary Figure 3 Hypothetical karyotypes of F1 hybrid clones, which could be obtained after crosses between Mchare cultivars (female parent) and *M. acuminata* ssp. *burmannicoides* ‘Calcutta 4’ (male parent). The chromosomes with translocations specific to Mchare genome are marked with a pink asterisk, and chromosomes with translocations specific to ‘Calcutta 4’ are marked with a green asterisk. Blue rectangles indicate karyotypes of F1 hybrid clones detected in our study.

Supplementary Figure 4 Genome structure of F1 hybrid clones gained after crosses of Mchare banana cultivars (female parent) and *M. acuminata* ssp. *burmannicoides* ‘Calcutta 4’ (male parent). Coverage ratio of alleles specific to Mchare genotypes (red dots) and to the *M. acuminata* ssp. *burmannicoides* ‘Calcutta 4’ (green dots) along 11 chromosomes of *M. acuminata* ssp*. malaccensis* ‘DH Pahang’ reference genome sequence.

## References

[B1] Amah D. Turner D. W. Gibbs J. Gil G. Swennen R. (2021). Overcoming the fertility crisis in bananas (*Musa* spp.). In: Achieving sustainable cultivation of bananas. Chapter 1, 1-50. Kema G. H. J. Drenth A. (eds.), *Achieving sustainable cultivation of bananas Volume 2: Germplasm and genetic improvement*. (Cambridge, UK: Burleigh Dodds Science Publishing). doi: 10.19103/AS.2020.0070.13

[B2] Bakry F. Horry J. P. (1992). Tetraploid hybrids from interploid 3x/2x crosses in cooking bananas. Fruits 47, 641–655.

[B3] Bandelt H. J. Dress A. W. M. (1992a). A canonical decomposition theory for metrics on a finite set, Adv. Math 92, 47–105. doi: 10.1016/0001-8708(92)90061-O

[B4] Bandelt H. J. Dress A. W. M. (1992b). Split decomposition: A new and useful approach to phylogenetic analysis of a distance data. Mol. Phylogenet. Evol. 1, 242–252. doi: 10.1016/1055-7903(92)90021-8 1342941

[B5] Batte M. Swennen R. Uwimana B. Akech V. Brown A. Tumuhimbise R.. (2019). Crossbreeding East African Highland Bananas: Lessons learnt relevant to the botany of the crop after 21 years of genetic enhancement. Front. Plant Sci. 10. doi: 10.3389/fpls.2019.00081 PMC6370977 30804965

[B6] Baurens F. C. Martin G. Hervouet C. Salmon F. Yohomé D. Ricci S.. (2019). Recombination and large structural variations shape interspecific edible banana genomes. Mol. Biol. Evol. 36, 97–111. doi: 10.1093/molbev/msy199 30403808 PMC6340459

[B7] Bayo S. J. Massawe V. Ndakidemi P. A. Venkataramana P. Mlaki A. Mduma H.. (2024). Pollen amount and viability in Mchare and selected wild (AA) banana (*Musa acuminata*) genotypes: Prospects for Breeding. HortSci 59, 632–638. doi: 10.21273/HORTSCI17608-23

[B8] Belser C. Baurens F.-C. Noel B. Martin G. Cruaud C. Istace B.. (2021). Telomere-to-telomere gapless chromosomes of banana using nanopore sequencing. Commun. Biol. 4, 1047. doi: 10.1038/s42003-021-02559-3 34493830 PMC8423783

[B9] Beránková D. Hřibová E. (2023). Bulked oligo-FISH for chromosome painting and chromosome barcoding. Methods Mol. Biol. 2672, 445–463. doi: 10.1007/978-1-0716-3226-0_27 37335493

[B10] Braz G. T. He L. Zhao H. Zhang T. Semrau K. Rouillard J. M.. (2018). Comparative oligo-FISH mapping: An efficient and powerful methodology to reveal karyotypic and chromosomal evolution. Genetics 208, 513–523. doi: 10.1534/genetics.117.300344 29242292 PMC5788518

[B11] Brown A. Tumuhimbise R. Amah D. Uwimana B. Nyine M. Mduma H.. (2017). “Bananas and Plantains (*Musa* spp.),” in Genetic Improvement of Tropical Crops (Springer, Cham).

[B12] Bryant D. Huson D. H. (2023). NeighborNet: improved algorithms and implementation. Front. Bioinf. 3. doi: 10.3389/fbinf.2023.1178600 PMC10548196 37799982

[B13] Chen S. Zhou Y. Chen Y. Gu J. (2018). fastp: an ultra-fast all-in-one FASTQ preprocessor. Bioinformatics 34, i884–i890. doi: 10.1093/bioinformatics/bty560 30423086 PMC6129281

[B14] Christelová P. De Langhe E. Hřibová E. Čížková J. Sardos J. Hušáková M.. (2017). Molecular and cytological characterization of the global Musa germplasm collection provides insight into the treasure of banana diversity. Biodivers. Conserv. 26, 801–824. doi: 10.1007/s10531-016-1273-9

[B15] Christelová P. Valárik M. Hřibová E. Van den Houwe I. Channeliere S. Roux N.. (2011). A platform for efficient genotyping in Musa using microsatellite markers. AoB Plants 2011, plr024. doi: 10.1093/aobpla/plr024 22476494 PMC3185971

[B16] Cordoba D. Jansen K. (2014). Same disease – different research strategies: Bananas and Black Sigatoga in Brazil and Colombia. Singap. J. Trop. Geogr. 35, 345–361. doi: 10.1111/sjtg.12072

[B17] De Bellaire L. L. Fouré E. Abadie C. Carlier J. (2010). Black leaf streak disease is challenging the banana industry. Fruits 65, 327–342. doi: 10.1051/fruits/2010034

[B18] De Langhe E. Hřibová E. Carpentier S. Doležel J. Swennen R. (2010). Did backcrossing contribute to the origin of hybrid edible bananas? Ann. Bot. 106, 849–857. doi: 10.1093/aob/mcq187 20858591 PMC2990659

[B19] D’Hont A. Denoeud F. Aury J. M. Baurens F. C. Carreel F. Garsmeur O.. (2012). The banana (Musa acuminata) genome and the evolution of monocotyledonous plants. Nature 488, 213–217. doi: 10.1038/nature11241 22801500

[B20] Dodds K. S. Simmonds N. W. (1948). Sterility and parthenocarpy in diploid hybrids of *Musa* . Heredity 2, 101–117. doi: 10.1038/hdy.1948.6 18863987

[B21] Doležel J. Doleželová M. Roux N. Van den Houwe I. (1998). A novel method to prepare slides for high resolution chromosome studies in Musa spp. Infomusa 7, 3–4.

[B22] Dumschott K. Schmidt M. H.-W. Chawla H. S. Snowdon R. Usadel B. (2020). Oxford Nanopore sequencing: new opportunities for plant genomics? J. Exp. Bot. 71, 5313–5322. doi: 10.1093/jxb/eraa263 32459850 PMC7501810

[B23] Dupouy M. Baurens F. C. Derouault P. Hervouet C. Cardi C. Cruaud C.. (2019). Two large reciprocal translocations characterized in the disease resistance-rich burmannica genetic group of Musa acuminata. Ann. Bot. 2019, 124:31–124329. doi: 10.1093/aob/mcz078 PMC6758587 31241133

[B24] FAO (2023). Banana market review – Preliminary results 2023 (Rome: FAO).

[B25] Farhat P. Mandáková T. Divíšek J. Kudoh H. German D. A. Lysak M. A. (2023). The evolution of the hypotetraploid Catolobus pendulus genome - the poorly known sister species of Capsella. Front. Plant. Sci. 14, 1165140. doi: 10.3389/fpls.2023.1165140 37223809 PMC10200890

[B26] Fauré S. Bakry F. Gonzalez L. D. (1993). “Cytogenetic studies of diploid bananas,” in Breeding banana and plantain for resistance to diseases and pests. Montpellier: CIRAD-FLHOR. Ed. Ganry J., 77–92. International symposium on genetic improvement of bananas for resistance to diseases and pests, Montpellier, France.

[B27] Goigoux S. Salmon F. Bakry F. (2013). Evaluation of pollen fertility of diploid and doubled-diploid clones of mlali and their potential use for banana breeding. ISHS Acta Hortic. 986, 195–204. doi: 10.17660/ActaHortic.2013.986.20

[B28] Han Y. Zhang T. Thammapichai P. Weng Y. Jinag J. (2015). Chromosome-specific painting in Cucumis species using bulked oligonucleotides. Genetics 200, 771–779. doi: 10.1534/genetics.115.177642 25971668 PMC4512542

[B29] Hippolyte I. Jenny C. Gardes L. Bakry F. Rivallan R. Pomies V.. (2012). Foundation characteristics of edible *Musa* triploids revealed from allelic distribution of SSR markers. Ann. Bot. 109, 937–951. doi: 10.1093/aob/mcs010 22323428 PMC3310492

[B30] Hou L. Xu M. Zhang T. Xu Z. Wang W. Zhang J.. (2018). Chromosome painting and its applications in cultivated and wild rice. BMC Plant Biol. 18, 110. doi: 10.1186/s12870-018-1325-2 29879904 PMC5991451

[B31] Huson D. H. Bryant D. (2006). Application of phylogenetic networks in evolutionary studies. Mol. Biol. Evol. 23, 254–267. doi: 10.1093/molbev/msj030 16221896

[B32] Ibarra-Laclette E. Lyons E. Hernández-Guzmán G. Pérez-Torres C. A. Carretero-Paulet L. Chang T. ,. H.. (2013). Architecture and evolution of a minute plant genome. Nature 498, 94–98. doi: 10.1038/nature12132 23665961 PMC4972453

[B33] Janssens S. B. Vandelook F. De Langhe E. Verstraete B. Smets E. Van den Houwe I.. (2016). Evolutionary dynamics and biogeography of Musaceae reveal a correlation between the diversification of the banana family and the geological and climatic history of Southeast Asia. New Phytol. 210, 1453–1465. doi: 10.1111/nph.13856 26832306 PMC5066818

[B34] Jiang J. (2019). Fluorescence *in situ* hybridization in plants: recent developments and future applications. Chromosome Res. 27, 153–165. doi: 10.1007/s10577-019-09607-z 30852707

[B35] Li H. (2013). Aligning sequence reads, clone sequences and assembly contigs with BWA-MEM. arXiv: Genomics. doi: 10.48550/arXiv.1303.3997

[B36] Liu X. Arshad R. Wang X. Li W. M. Zhou Y. Ge X.-J.. (2023). The phased telomere-to-telomere reference genome of *Musa acuminata*, a main contributor to banana cultivars. Sci. Data 10, 631. doi: 10.1038/s41597-023-02546-9 37716992 PMC10505225

[B37] Mandáková T. Lysak M. A. (2018). Post-polyploid diploidization and diversification through dysploid changes. Curr. Opin. Plant Biol. 42, 55–65. doi: 10.1016/j.pbi.2018.03.001 29567623

[B38] Mandáková T. Pouch M. Harmanová K. Zhan S. H. Mayrose I. Lysak M. A. (2017). Multispeed genome diploidization and diversification after an ancient allopolyploidization. Mol. Ecol. 22), 6445–6462. doi: 10.1111/mec.14379 29024107

[B39] Martin G. Baurens F. C. Hervouet C. Salmon F. Delos J. M. Labadie K.. (2020b). Chromosome reciprocal translocations have accompanied subspecies evolution in bananas. Plant J. 104, 1698–1711. doi: 10.1111/tpj.15031 33067829 PMC7839431

[B40] Martin G. Baurens F. C. Labadie K. Hervouet C. Salmon F. Marius F.. (2023b). Shared pedigree relationships and transmission of unreduced gametes in cultivated banana. Ann. Bot. 131, 1149–1161. doi: 10.1093/aob/mcad065 37267450 PMC10457027

[B41] Martin G. Cardi C. Sarah G. Ricci S. Jenny C. Fondi E.. (2020a). Genome ancestry mosaics reveal multiple and cryptic contributors to cultivated banana. Plant J. 102, 1008–1025. doi: 10.1111/tpj.14683 31930580 PMC7317953

[B42] Martin G. Carreel F. Coriton O. Hervouet C. Cardi C. Derouault P.. (2017). Evolution of the banana genome (*Musa acuminata*) is impacted by large chromosomal translocations. Mol. Biol. Evol. 34, 2140–2152. doi: 10.1093/molbev/msx164 28575404 PMC5850475

[B43] Martin G. Cottin A. Baurens F. C. Labadie K. Hervouet C. Salmon F.. (2023a). Interspecific introgression patterns reveal the origins of worldwide cultivated bananas in New Guinea. Plant J. 113, 802–818. doi: 10.1111/tpj.16086 36575919

[B44] McKenna A. Hanna M. Banks E. Sivachenko A. Cibulskis K. Kernytsky A.. (2010). The Genome Analysis Toolkit: a MapReduce framework for analyzing next-generation DNA sequencing data. Genome Res. 20, 1297–1303. doi: 10.1101/gr.107524.110 20644199 PMC2928508

[B45] Nyine M. Uwimana B. Swennen R. Batte M. Brown A. Christelová P.. (2017). Trait variation and genetic diversity in banana genomic selection training population. PloS One 12, e0178734. doi: 10.1371/journal.pone.0178734 28586365 PMC5460855

[B46] Ortiz R. (2013). Conventional banana and plantain breeding. Acta Hortic. 986, 77–194. doi: 10.17660/ActaHortic.2013.986.19

[B47] Ortiz R. Swennen R. (2014). From crossbreeding to biotechnology-facilitated improvement of banana and plantain. Biotechnol. Adv. 32, 158–169. doi: 10.1016/j.biotechadv.2013.09.010 24091289

[B48] Perrier X. Bakry F. Carreel F. Jenny F. Horry J. P. Lebot V.. (2009). Combining biological approaches to shed light on the evolution of edible bananas. Ethnobot. Res. Appl. 7, 199–216. doi: 10.17348/era.7.0.199-216

[B49] Perrier X. De Langhe E. Donohue M. Lentfer C. J. Vrydaghs L. Bakry F.. (2011). Multidisciplinary perspectives on banana (*Musa* spp.) domestication. Proc. Natl. Acd. Sci. U.S.A. 108, 11311–11318. doi: 10.1073/pnas.1102001108 PMC3136277 21730145

[B50] Perrier X. Jenny C. Bakry F. Karamura D. Kitavi M. Dubois C.. (2019). East African diploid and triploid bananas: a genetic complex transported from South-East Asia. Ann. Bot. 123, 19–36. doi: 10.1093/aob/mcy156 30247503 PMC6344093

[B51] Pucker B. Irisarri I. de Vries J. Xu B. (2022). Plant genome sequence assembly in the era of long reads: Progress, challenges and future directions. Quant. Plant Biol. 3, e5. doi: 10.1017/qpb.2021.18 37077982 PMC10095996

[B52] Raboin L. M. Careel F. Noyer J. L. Baurens F. C. Horry J. P. Du Montcel H. T.. (2005). Diploid ancestors of triploid export banana cultivars: Molecular identification of *2n* restitution gamete donor and *n* gamete donors. Mol. Breed. 16, 333–341. doi: 10.1007/s11032-005-2452-7

[B53] Salse J. (2016). Deciphering the evolutionary interplay between subgenomes following polyploidy: A paleogenomics approach in grasses. Am. J. Bot. 103, 1167–1174. doi: 10.3732/ajb.1500459 27425631

[B54] Sardos J. Breton C. Perrier X. Van den Houwe I. Carpentier S. Paofa J.. (2022). Hybridization, missing wild ancestors and the domestication of cultivated diploid bananas. Front. Plant Sci. 13. doi: 10.3389/fpls.2022.969220 PMC9586208 36275535

[B55] Sardos J. Perrier X. Doležel J. Hřibová E. Christelová P. Van den Houwe I.. (2016a). DArT whole genome profiling provides insights on evolution and taxonomy of edible banana (*Musa* spp.). Ann. Bot. 118, 1269–1278. doi: 10.1093/aob/mcw170 27590334 PMC5155597

[B56] Schubert I. Vu G. T. H. (2016). Genome stability and evolution: attempting a holistic view. Trends Plant Sci. 21, 749–757. doi: 10.1016/j.tplants.2016.06.003 27427334

[B57] Shepherd K. (1999). Cytogenetics of the genus *Musa* (Montpellier: International Network for the Improvement of Bananas and Plantain).

[B58] Simmonds N. W. Shepherd K. (1955). The taxonomy and origin of the cultivated bananas. Bot. J. Linn. Soc 55, 302–312. doi: 10.1111/j.1095-8339.1955.tb00015.x

[B59] Šimoníková D. Němečková A. Čížková J. Brown A. Swennen R. Doležel J.. (2020). Chromosome painting in cultivated bananas and their wild relatives (*Musa* spp.) reveals differences in chromosome structure. Int. J. Mol. Sci. 21, 7915. doi: 10.3390/ijms21217915 33114462 PMC7672600

[B60] Šimoníková D. Němečková A. Karafiátová M. Uwimana B. Swennen R. Doležel J.. (2019). Chromosome painting facilitates anchoring reference genome sequence to chromosomes *in situ* and integrated karyotyping in banana (*Musa* spp.). Front. Plant Sci. 10. doi: 10.3389/fpls.2019.01503 PMC6879668 31824534

[B61] Stull G. W. Pham K. K. Soltis P. S. Soltis D. E. (2023). Deep reticulation: the long legacy of hybridization in vascular plant evolution. Plant J. 114, 743–766. doi: 10.1111/tpj.16142 36775995

[B62] Tomekpe K. Jenny C. Escalant J. V. (2004). A review of conventional improvement strategies for *Musa* . Infomusa 13, 2–6.

[B63] Wang X. Morton J. A. Pellicer J. Leitch I. J. Leitch A. R. (2021). Genome downsizing after polyploidy: mechanisms, rates and selection pressures. Plant J. 107, 1003–1015. doi: 10.1111/tpj.15363 34077584

[B64] Wendel J. F. (2000). Genome evolution in polyploids. Plant Mol. Biol. 42, 225–249. doi: 10.1023/A:1006392424384 10688139

[B65] Yu F. Zhao X. Chai J. Ding X. Li X. Huang Y.. (2022). Chromosome-specific painting unveils chromosomal fusions and distinct allopolyploid species in the *Saccharum* complex. New Phytol. 233, 1953–1965. doi: 10.1111/nph.17905 34874076

