# Supplementary material for: Striking variation in chromosome structure within Musa acuminata subspecies, diploid cultivars, and F1 diploid hybrids
Source: Front Plant Sci. 2024 Jul 4;15:1387055. doi: 10.3389/fpls.2024.1387055 (PMC11255410; doi:10.3389/fpls.2024.1387055)

A

cv. 'Vudu Beo' (ITC 1211)

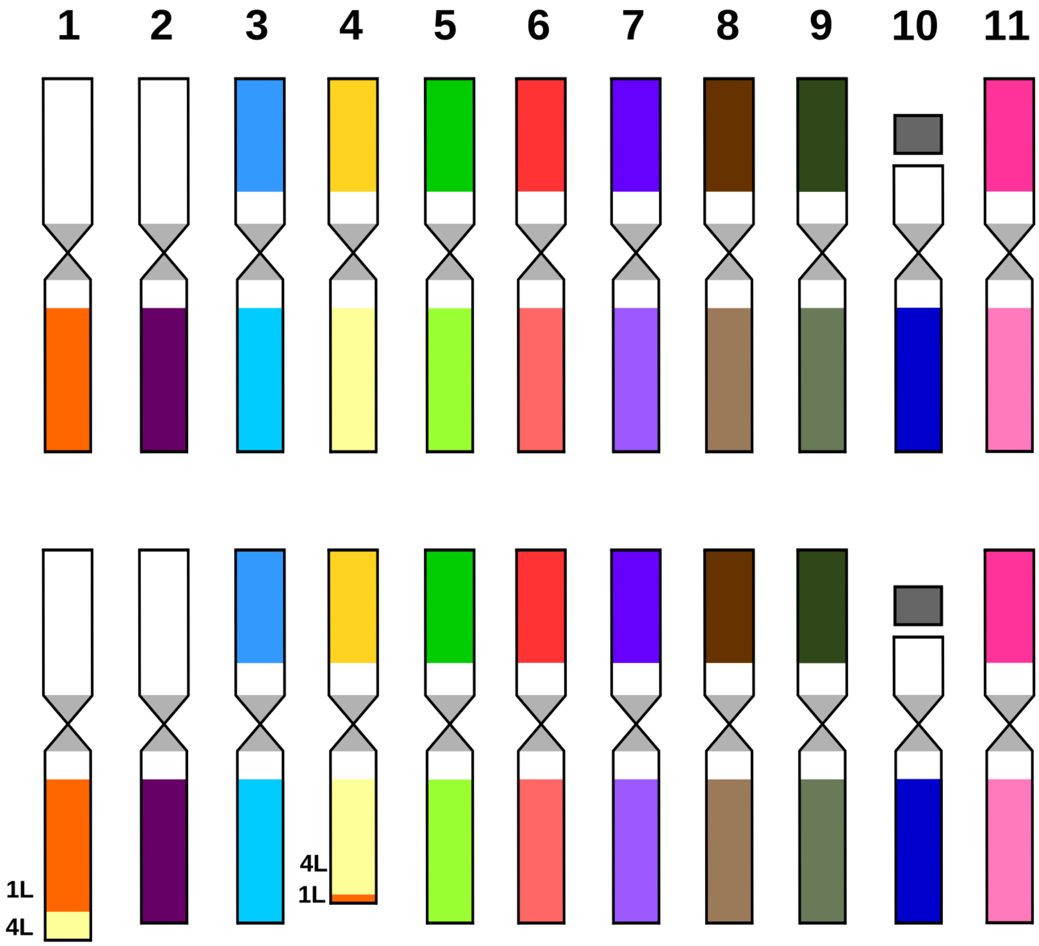

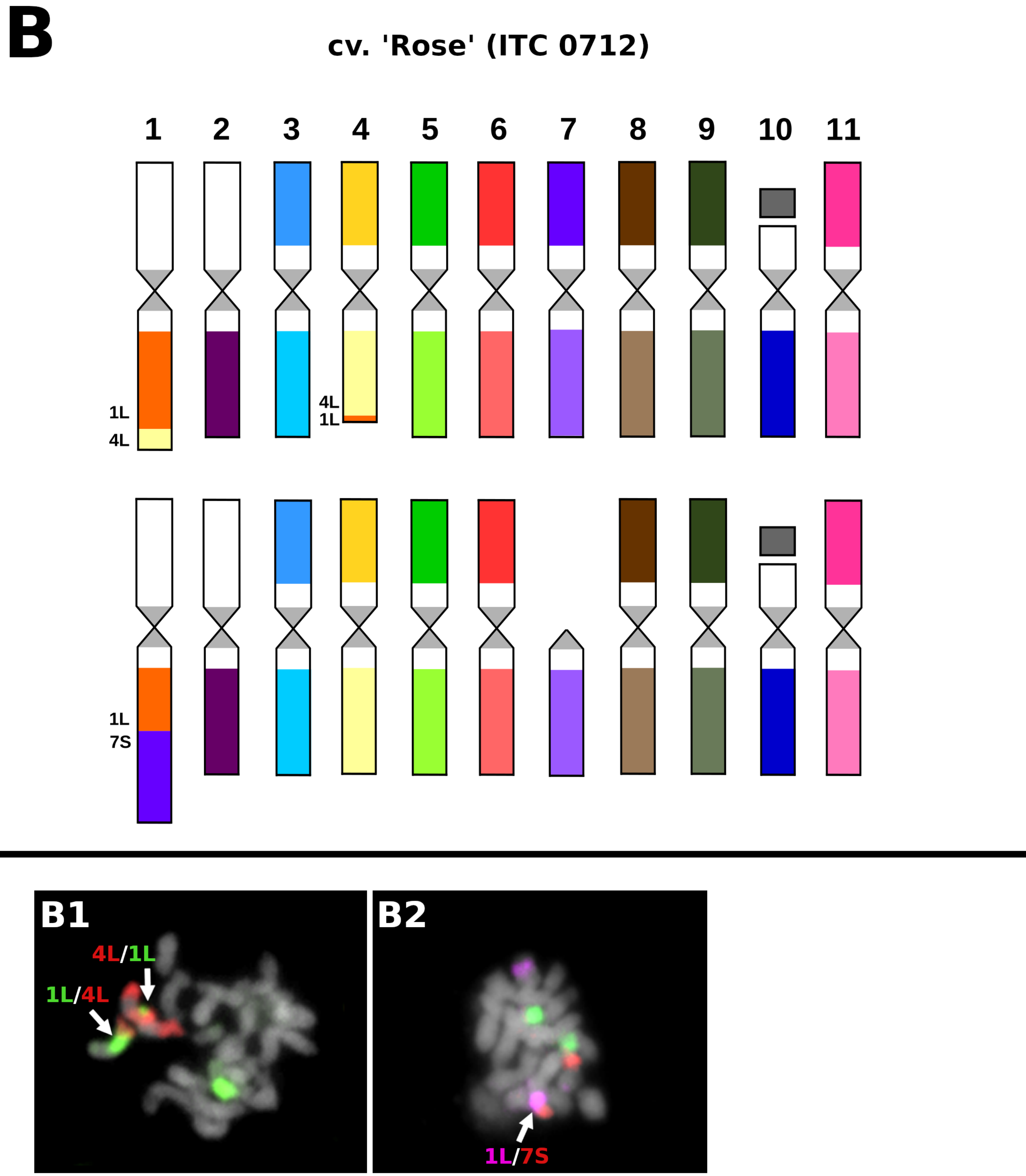

Examples of oligo painting FISH on mitotic chromosomes of diploid cv. 'Rose' (ITC0712): B1) probes for chromosomes 1L (green) and 4L (red); B2) probes for chromosomes 1l (purple), 7S (red), and 7L (green). Chromosomes were counterstained with DAPI (light grey pseudocolor). Arrows point to translocations.

C

M. acuminata ssp. _zebrina_ 'Zebrina' (ITC 1139)
cv. 'Mai'a hapai (subgr. Sucrier; ITC 1172)

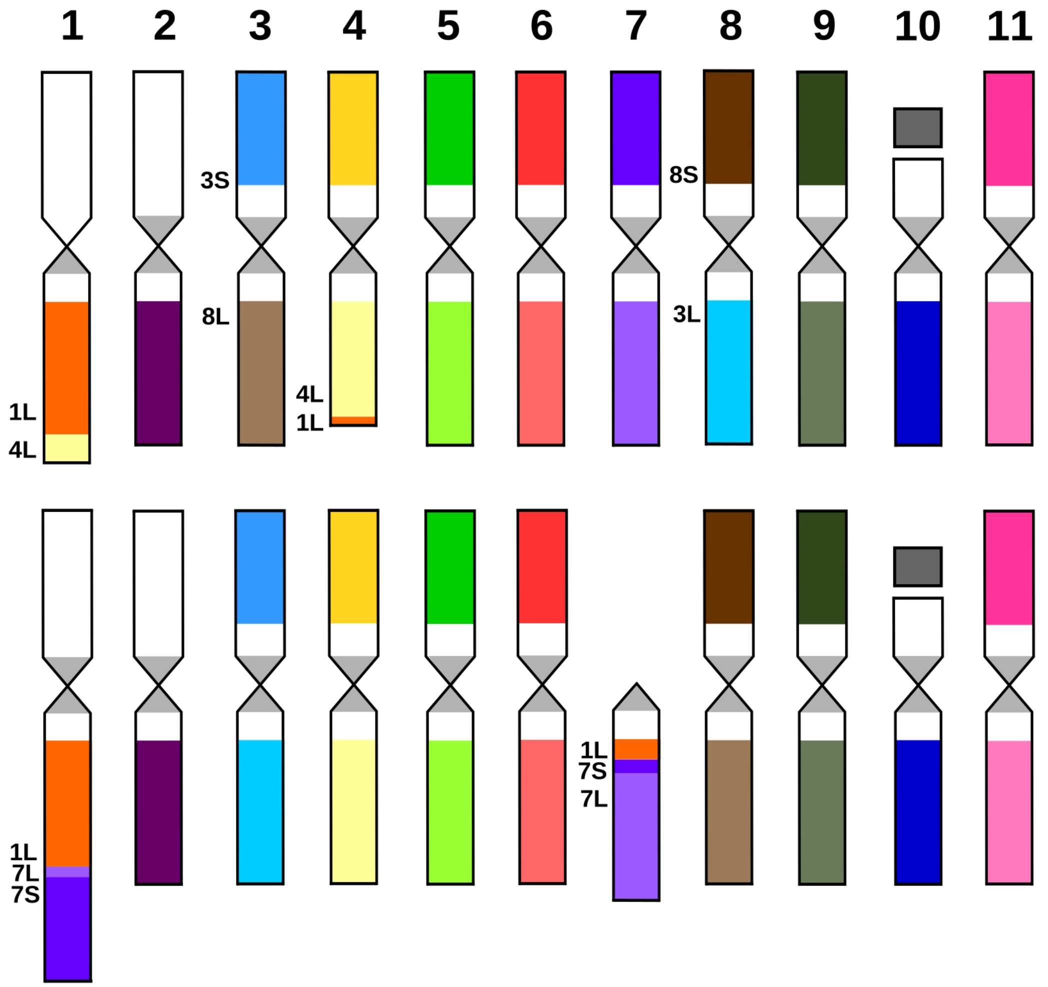

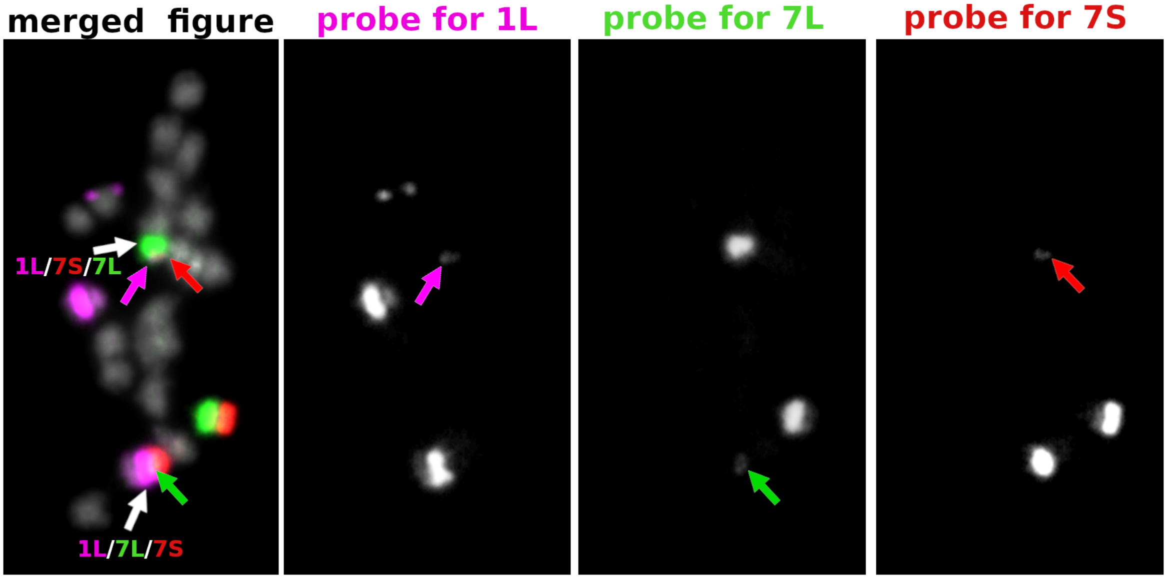

Minor signals of the probes on translocated chromosomes are marked by arrows in corresponding colors.

D

Musa acuminata ssp. *siamea* 'Khae (Phrae)' (ITC 0660)

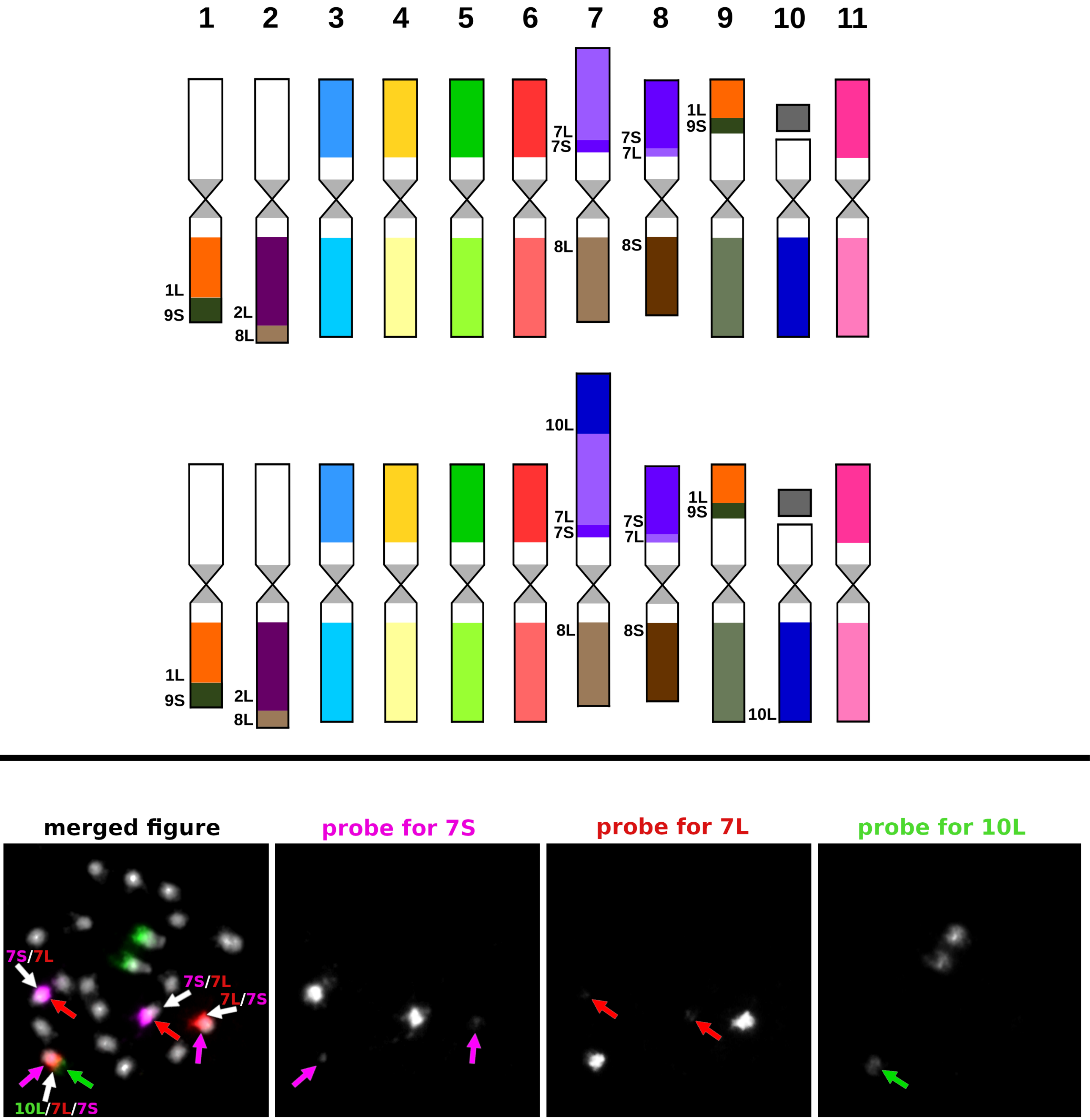

Minor signals of the probes on translocated chromosomes are marked by arrows in corresponding colors.

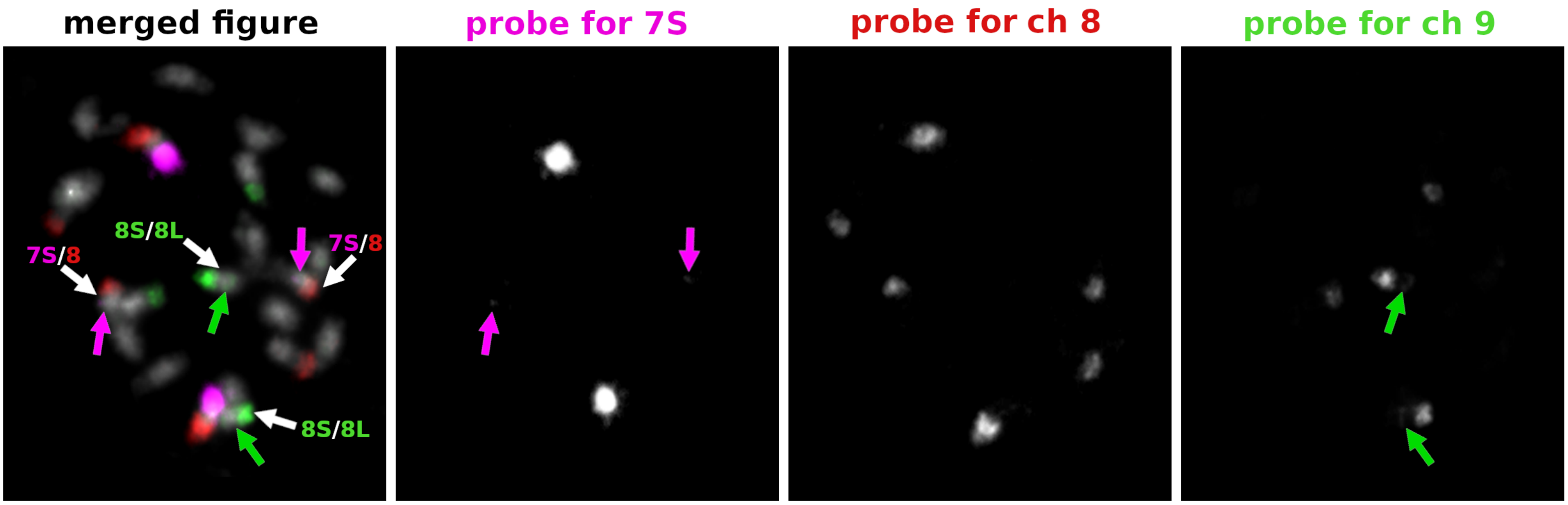

Minor signals of the probes on translocated chromosomes are marked by arrows in corresponding colors.

E

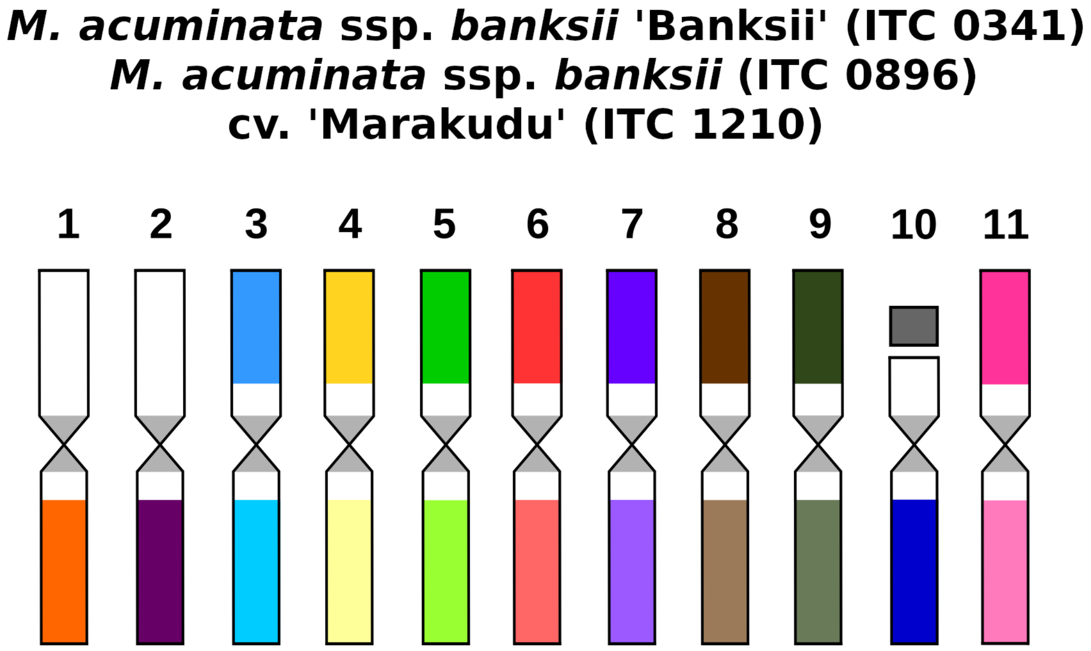
M. acuminata ssp. *banksii* 'Banksii' (ITC 0341)
M. acuminata ssp. *banksii* (ITC 0896)
cv. 'Marakudu' (ITC 1210)
1
2
3
4
5
6
7
8
9
10
11

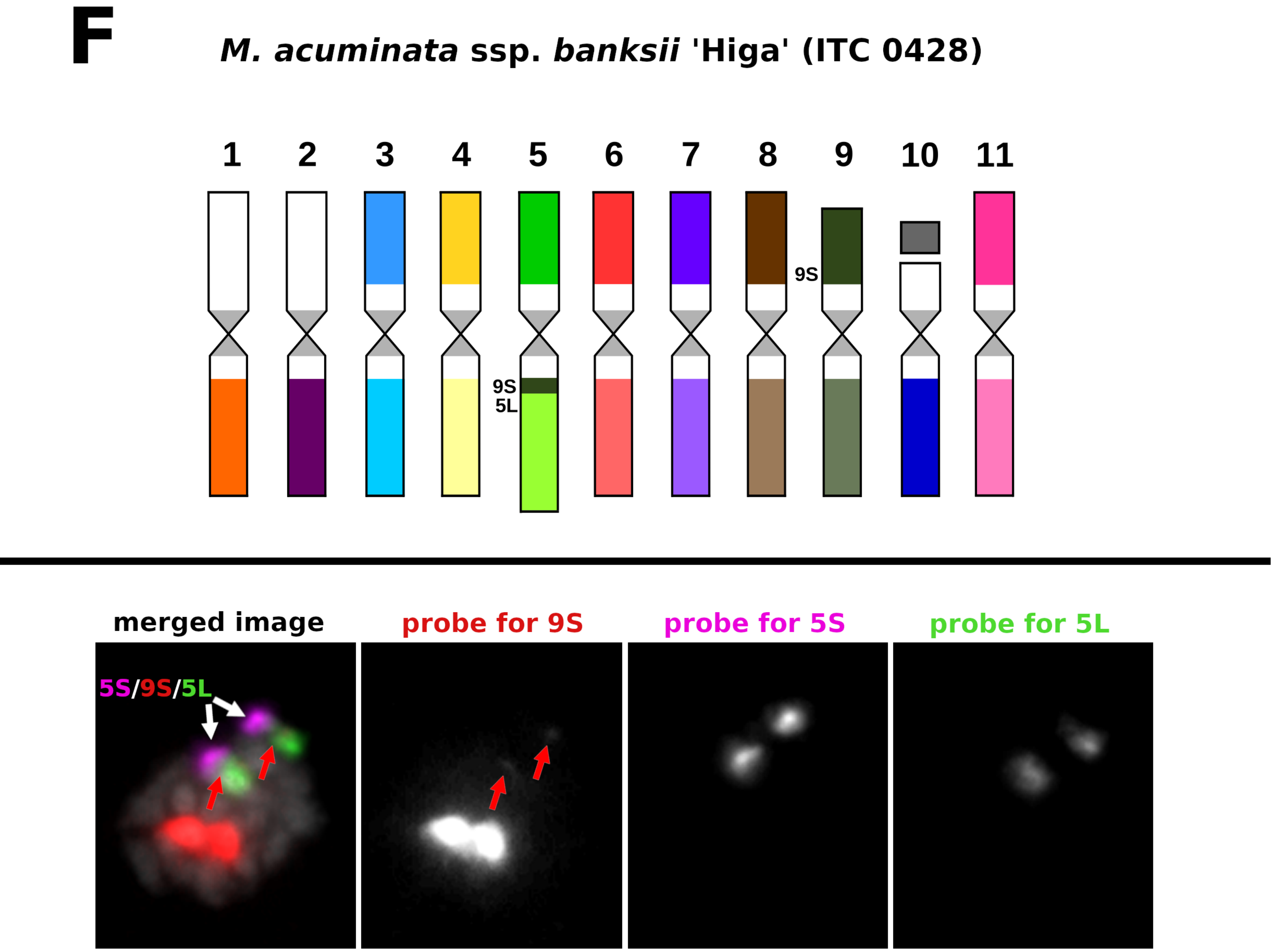

Minor signals of the probes on translocated chromosomes are marked by arrows in corresponding colors.

G

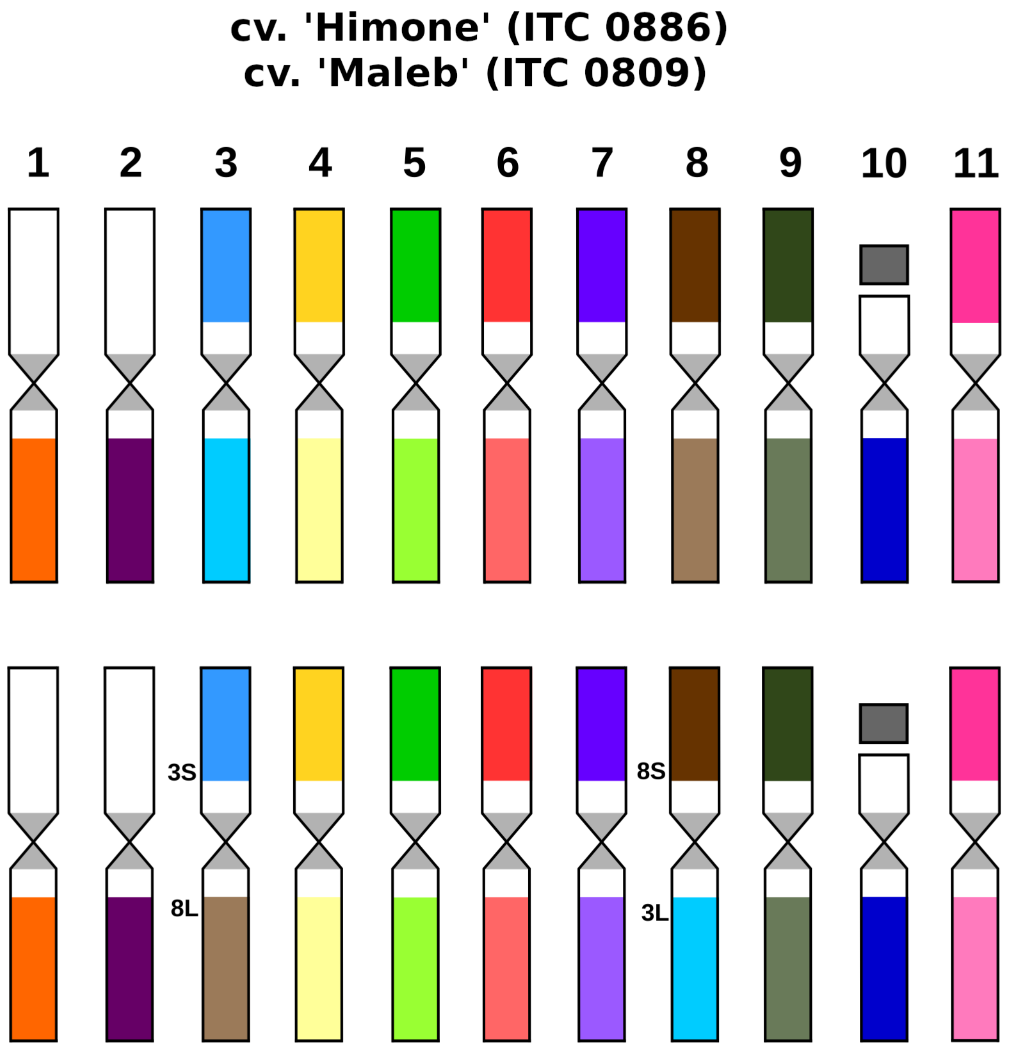
cv. 'Himone' (ITC 0886)
cv. 'Maleb' (ITC 0809)
1
2
3
4
5
6
7
8
9
10
11
3S
8L
8S
3L

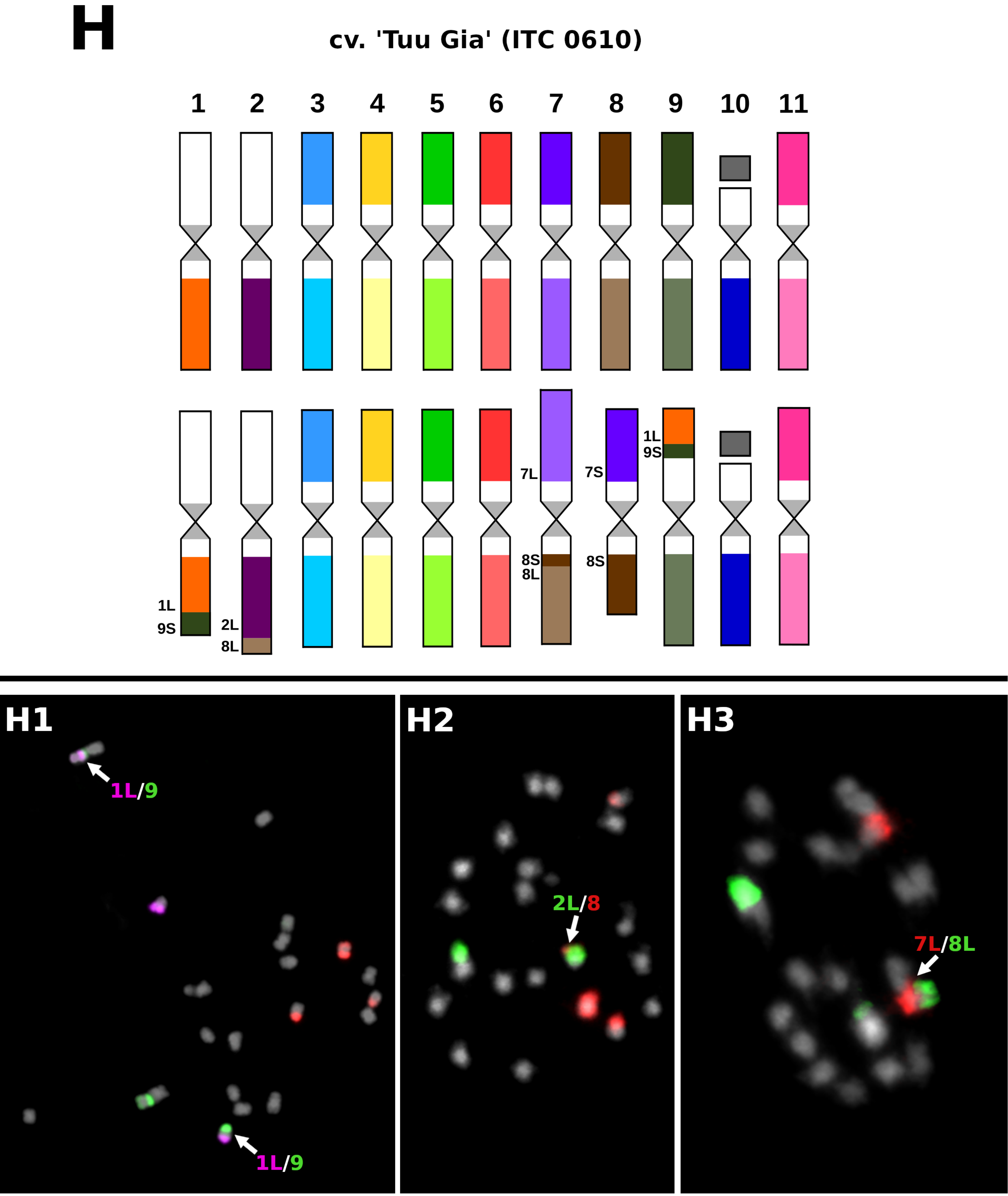

Examples of oligo painting FISH on mitotic chromosomes of 'Tuu Gia':H1) probes for chromosomes 9 (green), 7 (red), and 1 (pink); H2) probes for chromosomes 2L (green) and 8 (red), and H3) probes for chromosomes 7L (red) and 8L (green). Chromosomes were counterstained in DAPI (light grey pseudocolor). Arrows point to translocations.

I

cv. 'Mchare mlelembo' (subgr. Mchare)
cv. 'Mchare laini' (subgr. Mchare)
cv. 'Kahuti' (subgr. Mchare)

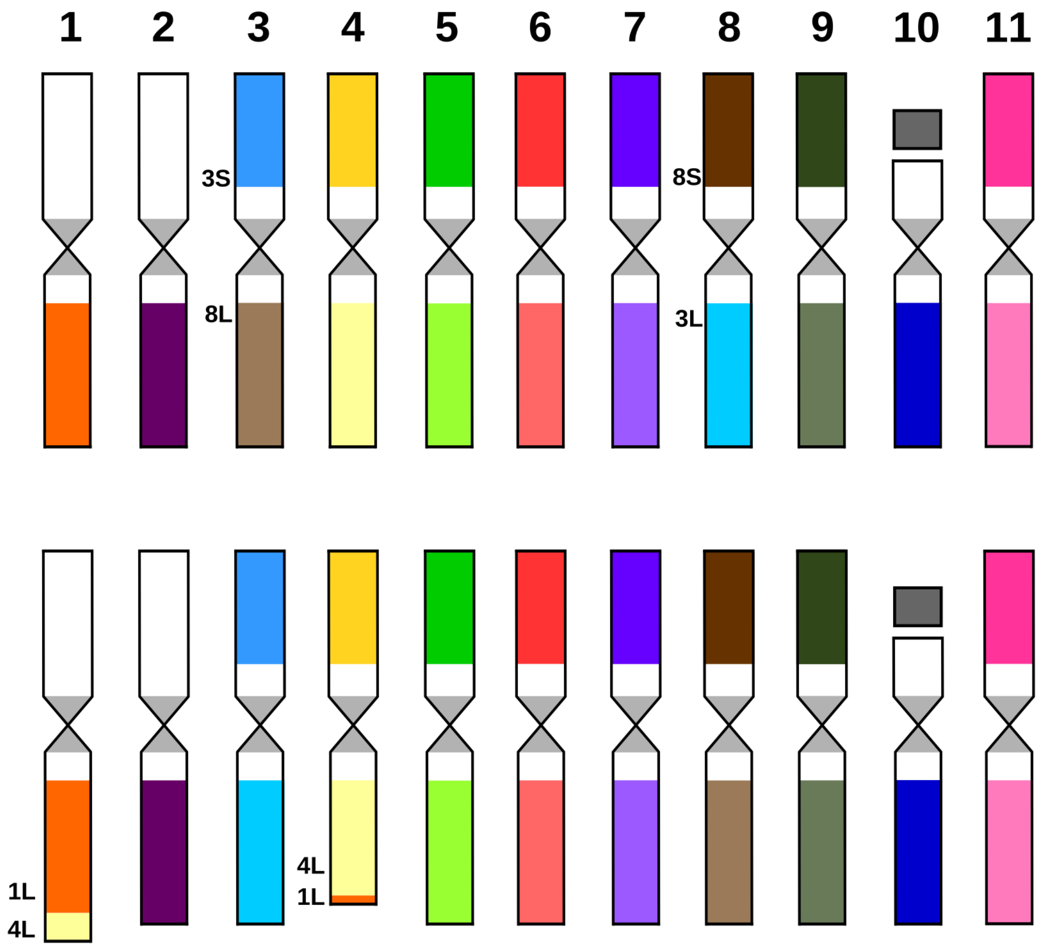

J

F1 hybrid clone 'T.2269-1'
('Huti white' x 'Calcutta 4')

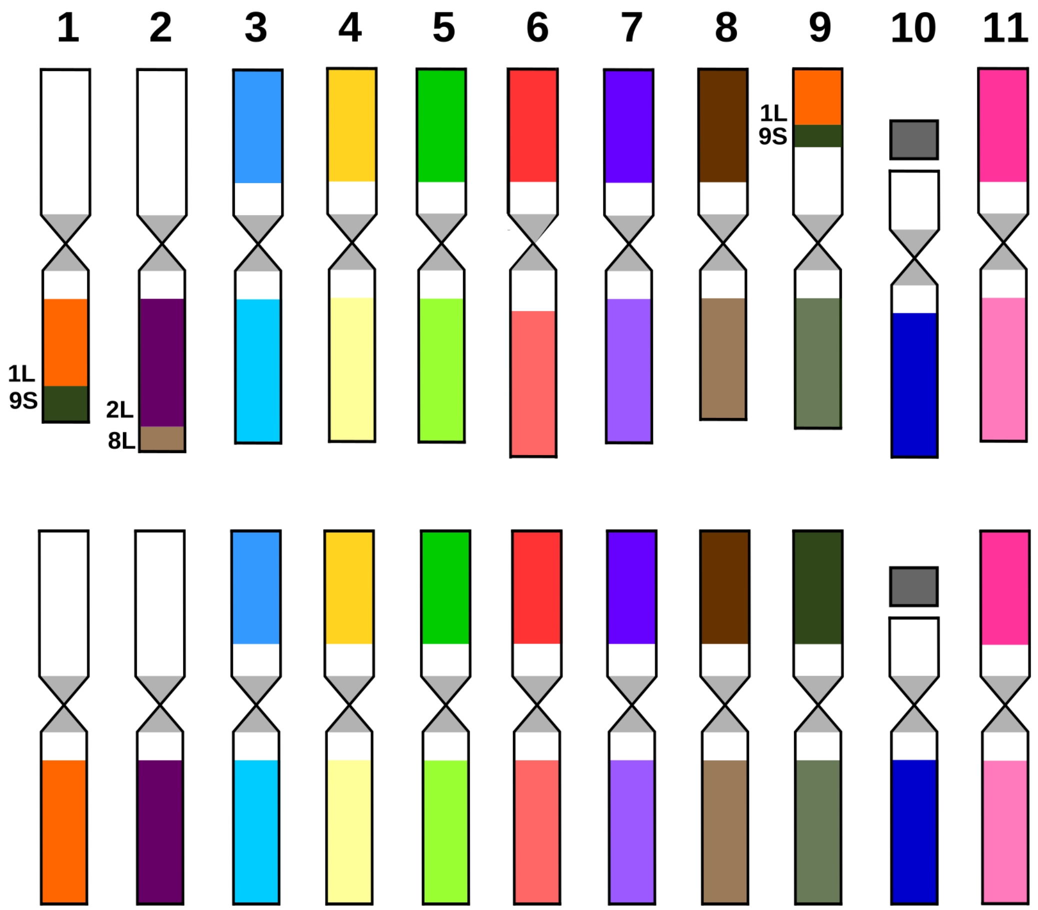

Supplement: Supplementary Figure 2 — Idiograms and short translocations (duplications) observed by oligo painting FISH in genomes of analyzed M. acuminata species and its edible banana clones. (A) Idiogram of cv. ‘Vudu Beo’ ITC1211; (B) Idiogram of cv. ‘Rose’ ITC0712 and oligo painting FISH with the probes for long arms of chromosomes 1 and 4, and oligo painting FISH with the probes for long arm of chromosome 1, short arm of chromosome 7 and long arm of chromosome 7; (C) Idiogram of M. acuminata ssp. zebrina ITC1139 and cv. ‘Mai’a hapai’ ITC1172, and oligo painting FISH with the probes for long arm of chromosome 1, and probes for short and long arms of chromosome 7; (D) Idiogram of M. acuminata ssp. siamea ‘Khae (Phrae)’ ITC0660, and oligo painting FISH with the probes for short arm of chromosome 7, long arm of chromosome 7 and long arm of chromosome 10L, and oligo painting FISH with the probes for short arm of chromosome 7 and probes for entire chromosomes 8 and 9; (E) Idiogram of M. acuminata ssp. banksii ITC0341 and ITC 0896, and cv. ‘Marakudu’ ITC1210; (F) Idiogram of M. acuminata ssp. banksii ‘Higa’ ITC0428 and oligo painting FISH with the probes for short arm of chromosome 9, short arm of chromosome 5 and long arm of chromosome 5; (G) Idiogram of cv. ‘Himone’ ITC0886 and cv. ‘Maleb’ ITC0809; (H) Idiogram of cv. ‘Tuu Gia’ ITC0610 and oligo painting FISH with the probes for long arm of chromosome 1 and entire chromosome, oligo painting FISH with the probes for long arm of chromosome 2 and entire chromosome 8, and oligo painting FISH with the probes long arms of chromosomes 7 and 8; (I) Idiogram of cv. ‘Mchare mlelembo’, cv. ‘Mchare laini’ and cv. ‘Kahuti’; and idiogram of F1 hybrid clone gained after crosses of Mchare banana cultivars (female parent) and M. acuminata ssp. burmannicoides ‘Calcutta 4’ (male parent): (J) ‘T2269–1’ (2n=2x=22). [file DataSheet_2.pdf]
