# Supplementary figures and images for: Striking variation in chromosome structure within Musa acuminata subspecies, diploid cultivars, and F1 diploid hybrids
Source: Front Plant Sci. 2024 Jul 4;15:1387055. doi: 10.3389/fpls.2024.1387055 (PMC11255410; doi:10.3389/fpls.2024.1387055)

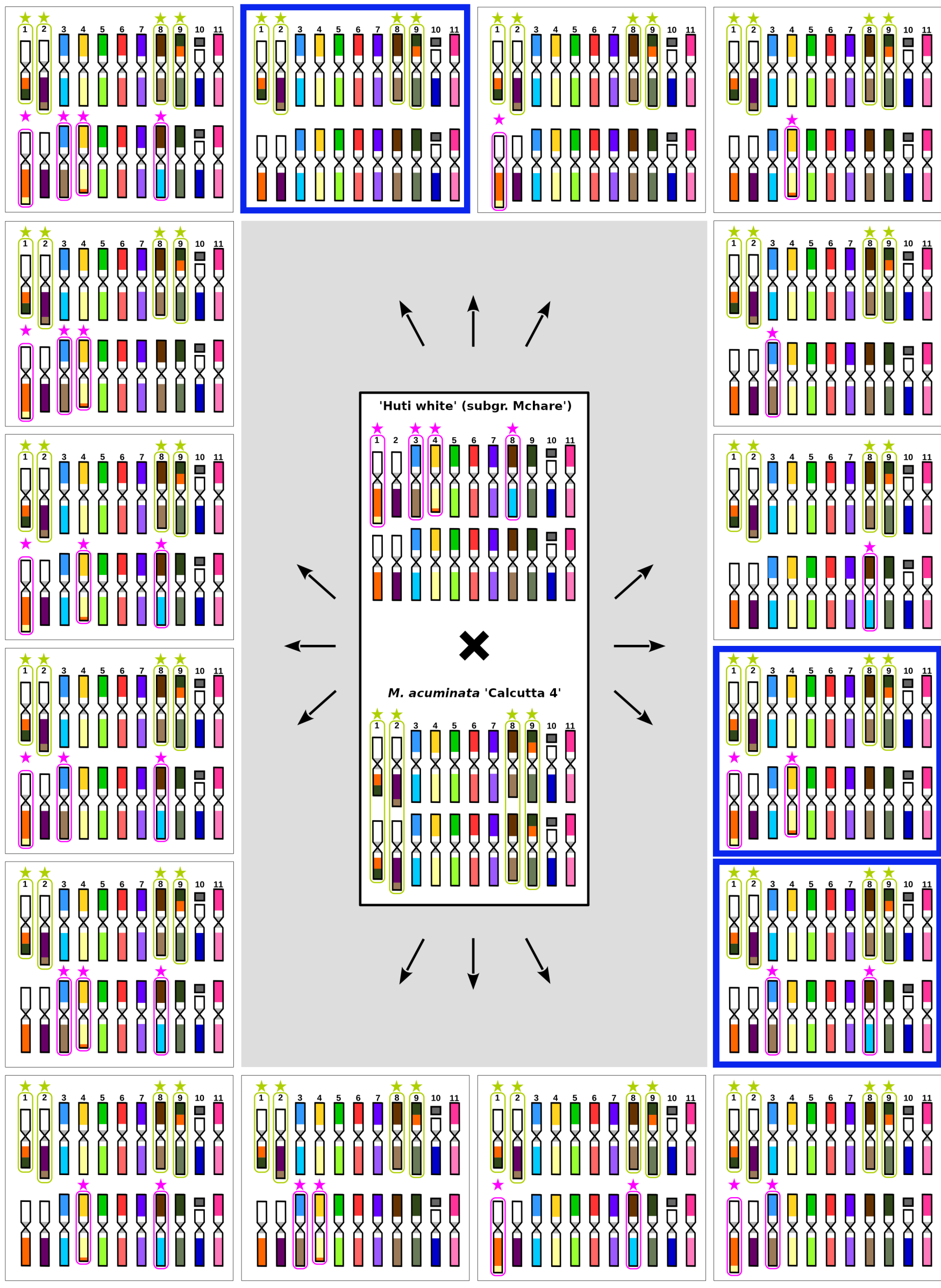

Supplement: Supplementary Figure 3 — Hypothetical karyotypes of F1 hybrid clones, which could be obtained after crosses between Mchare cultivars (female parent) and M. acuminata ssp. burmannicoides ‘Calcutta 4’ (male parent). The chromosomes with translocations specific to Mchare genome are marked with a pink asterisk, and chromosomes with translocations specific to ‘Calcutta 4’ are marked with a green asterisk. Blue rectangles indicate karyotypes of F1 hybrid clones detected in our study. [file DataSheet_3.pdf]
