# Supplementary material for: Striking variation in chromosome structure within Musa acuminata subspecies, diploid cultivars, and F1 diploid hybrids
Source: Front Plant Sci. 2024 Jul 4;15:1387055. doi: 10.3389/fpls.2024.1387055 (PMC11255410; doi:10.3389/fpls.2024.1387055)
Supplement: Supplementary Figure 4 — Genome structure of F1 hybrid clones gained after crosses of Mchare banana cultivars (female parent) and M. acuminata ssp. burmannicoides ‘Calcutta 4’ (male parent). Coverage ratio of alleles specific to Mchare genotypes (red dots) and to the M. acuminata ssp. burmannicoides ‘Calcutta 4’ (green dots) along 11 chromosomes of M. acuminata ssp. malaccensis ‘DH Pahang’ reference genome sequence. [file DataSheet_4.pdf]

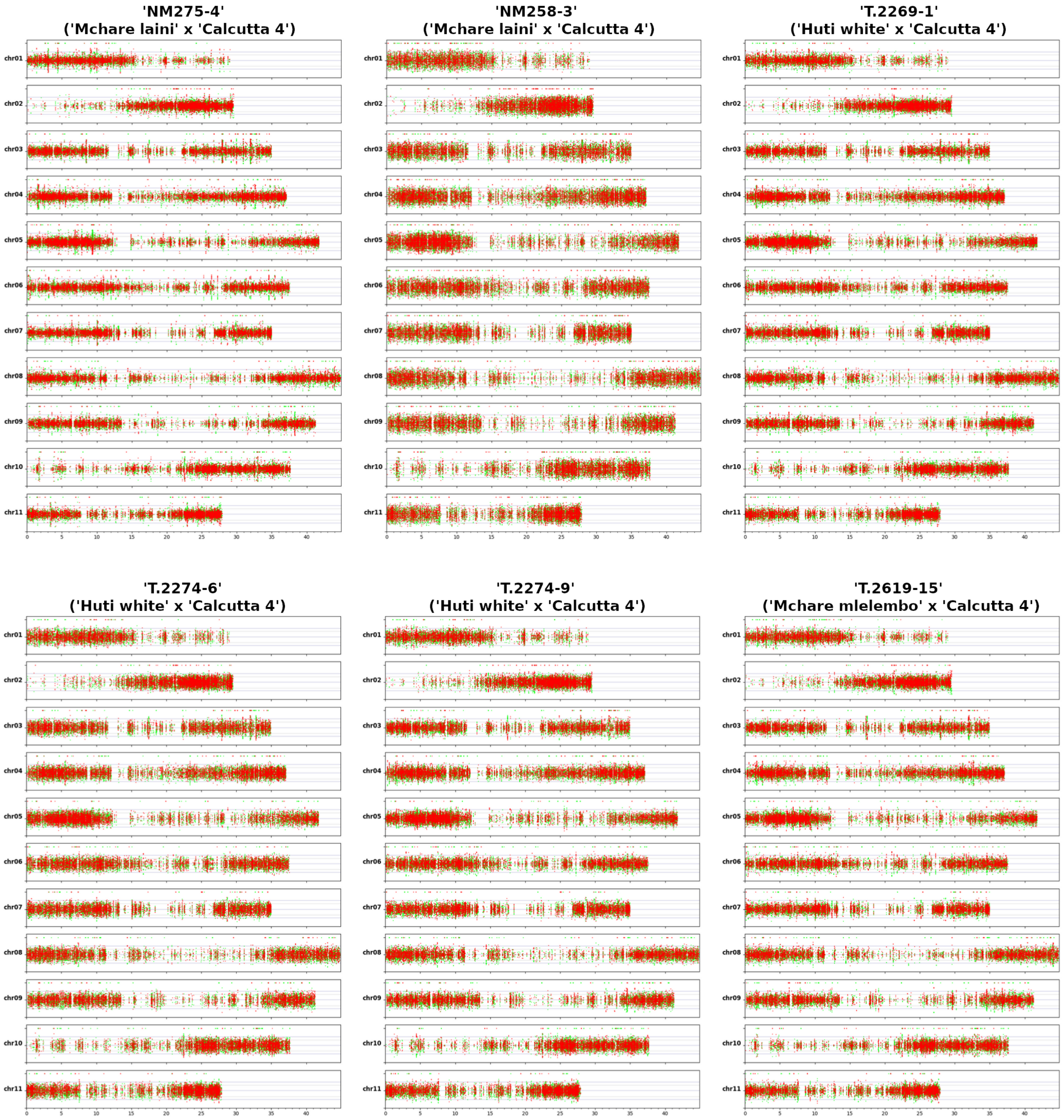
'NM275-4'
('Mchare laini' x 'Calcutta 4')
'NM258-3'
('Mchare laini' x 'Calcutta 4')
'T.2269-1'
('Huti white' x 'Calcutta 4')
'T.2274-6'
('Huti white' x 'Calcutta 4')
'T.2274-9'
('Huti white' x 'Calcutta 4')
'T.2619-15'
('Mchare mlelembo' x 'Calcutta 4')
chr01
chr02
chr03
chr04
chr05
chr06
chr07
chr08
chr09
chr10
chr11
0
5
10
15
20
25
30
35
40
