# Supplementary material for: Striking variation in chromosome structure within Musa acuminata subspecies, diploid cultivars, and F1 diploid hybrids
Source: Front Plant Sci. 2024 Jul 4;15:1387055. doi: 10.3389/fpls.2024.1387055 (PMC11255410; doi:10.3389/fpls.2024.1387055)
Supplement: Supplementary Figure 1 — Neighbor-Net analysis of Musa accessions and F1 hybrids between Mchare and M. acuminata ssp. burmannicoides ‘Calcutta 4’ performed by SplitsTree. Accessions representing different M. acuminata subspecies and group of cultivars are depicted in colors: banksii ssp. in red; malaccensis ssp. in violet; burmannica/burmannicoides/siamea in blue; zebrina in green; microcarpa in pink; Mchare genotypes in light blue; Sucrier genotypes in orange; other analyzed banana AA cultivars in black; and F1 progeny of Mchare and M. acuminata ssp. burmannicoides ‘Calcutta 4’ in brown. The accessions which were used for chromosome painting are depicted as squared nodes in the neighbor net tree. [file DataSheet_1.pdf]

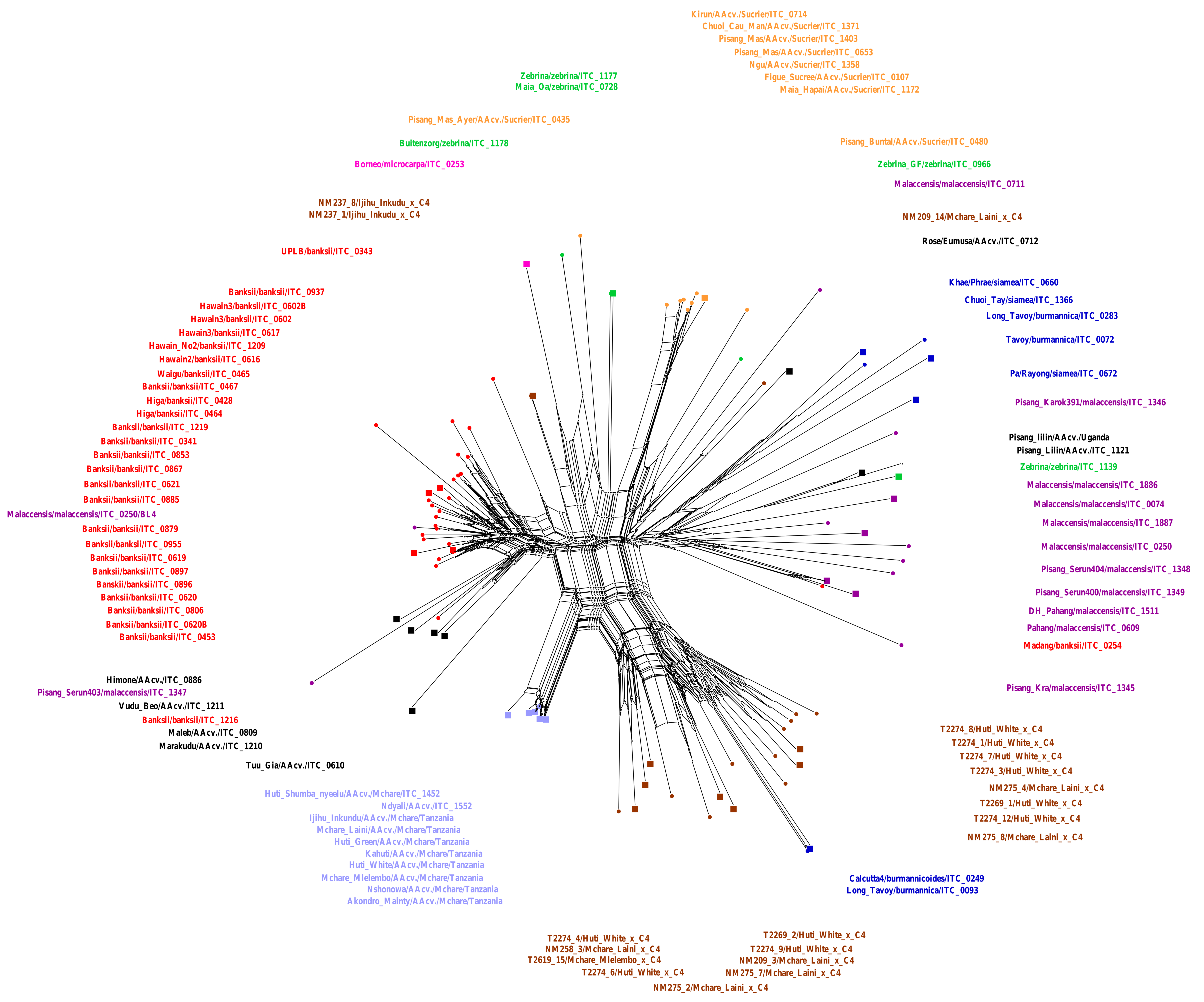

Kirun/AAcv./Sucrier/ITC_0714
Chuoi_Cau_Man/AAcv./Sucrier/ITC_1371
Pisang_Mas/AAcv./Sucrier/ITC_1403
Pisang_Mas/AAcv./Sucrier/ITC_0653
Ngu/AAcv./Sucrier/ITC_1358
Figue_Sucree/AAcv./Sucrier/ITC_0107
Maia_Hapai/AAcv./Sucrier/ITC_1172
Zebrina/zebrina/ITC_1177
Maia_Oa/zebrina/ITC_0728
Pisang_Mas_Ayer/AAcv./Sucrier/ITC_0435
Buitenzorg/zebrina/ITC_1178
Borneo/microcarpa/ITC_0253
Pisang_Buntal/AAcv./Sucrier/ITC_0480
Zebrina_GF/zebrina/ITC_0966
Malaccensis/malaccensis/ITC_0711
NM237_8/Ijihu_Inkudu_x_C4
NM237_1/Ijihu_Inkudu_x_C4
NM209_14/Mchare_Laini_x_C4
Rose/Eumusa/AAcv./ITC_0712
UPLB/banksii/ITC_0343
Banksii/banksii/ITC_0937
Hawain3/banksii/ITC_0602B
Hawain3/banksii/ITC_0602
Hawain3/banksii/ITC_0617
Hawain_No2/banksii/ITC_1209
Hawain2/banksii/ITC_0616
Waigu/banksii/ITC_0465
Banksii/banksii/ITC_0467
Higa/banksii/ITC_0428
Higa/banksii/ITC_0464
Banksii/banksii/ITC_1219
Banksii/banksii/ITC_0341
Banksii/banksii/ITC_0853
Banksii/banksii/ITC_0867
Banksii/banksii/ITC_0621
Banksii/banksii/ITC_0885
Malaccensis/malaccensis/ITC_0250/BL4
Banksii/banksii/ITC_0879
Banksii/banksii/ITC_0955
Banksii/banksii/ITC_0619
Banksii/banksii/ITC_0897
Banskii/banksii/ITC_0896
Banksii/banksii/ITC_0620
Banksii/banksii/ITC_0806
Banksii/banksii/ITC_0620B
Banksii/banksii/ITC_0453
Khae/Phrae/siamea/ITC_0660
Chuoi_Tay/siamea/ITC_1366
Long_Tavoy/burmannica/ITC_0283
Tavoy/burmannica/ITC_0072
Pa/Rayong/siamea/ITC_0672
Pisang_Karok391/malaccensis/ITC_1346
Pisang_lilin/AAcv./Uganda
Pisang_Lilin/AAcv./ITC_1121
Zebrina/zebrina/ITC_1139
Malaccensis/malaccensis/ITC_1886
Malaccensis/malaccensis/ITC_0074
Malaccensis/malaccensis/ITC_1887
Malaccensis/malaccensis/ITC_0250
Pisang_Serun404/malaccensis/ITC_1348
Pisang_Serun400/malaccensis/ITC_1349
DH_Pahang/malaccensis/ITC_1511
Pahang/malaccensis/ITC_0609
Madang/banksii/ITC_0254
Pisang_Kra/malaccensis/ITC_1345
Himone/AAcv./ITC_0886
Pisang_Serun403/malaccensis/ITC_1347
Vudu_Beo/AAcv./ITC_1211
Banksii/banksii/ITC_1216
Maleb/AAcv./ITC_0809
Marakudu/AAcv./ITC_1210
Tuu_Gia/AAcv./ITC_0610
Huti_Shumba_nyeelu/AAcv./Mchare/ITC_1452
Ndyali/AAcv./ITC_1552
Ijihu_Inkundu/AAcv./Mchare/Tanzania
Mchare_Laini/AAcv./Mchare/Tanzania
Huti_Green/AAcv./Mchare/Tanzania
Kahuti/AAcv./Mchare/Tanzania
Huti_White/AAcv./Mchare/Tanzania
Mchare_Mlelembo/AAcv./Mchare/Tanzania
Nshonowa/AAcv./Mchare/Tanzania
Akondro_Mainty/AAcv./Mchare/Tanzania
T2274_8/Huti_White_x_C4
T2274_1/Huti_White_x_C4
T2274_7/Huti_White_x_C4
T2274_3/Huti_White_x_C4
NM275_4/Mchare_Laini_x_C4
T2269_1/Huti_White_x_C4
T2274_12/Huti_White_x_C4
NM275_8/Mchare_Laini_x_C4
Calcutta4/burmannicoides/ITC_0249
Long_Tavoy/burmannica/ITC_0093
T2274_4/Huti_White_x_C4
NM258_3/Mchare_Laini_x_C4
T2619_15/Mchare_Mlelembo_x_C4
T2274_6/Huti_White_x_C4
NM275_2/Mchare_Laini_x_C4
T2269_2/Huti_White_x_C4
T2274_9/Huti_White_x_C4
NM209_3/Mchare_Laini_x_C4
NM275_7/Mchare_Laini_x_C4
